# NAC Transcription Factor Family Regulation of Fruit Ripening and Quality: A Review

**DOI:** 10.3390/cells11030525

**Published:** 2022-02-02

**Authors:** Gang-Shuai Liu, Hong-Li Li, Donald Grierson, Da-Qi Fu

**Affiliations:** 1 Laboratory of Fruit Biology, College of Food Science & Nutritional Engineering, China Agricultural University, Beijing 100083, China; lgsliugangshuai@cau.edu.cn (G.-S.L.); lhl199767@cau.edu.cn (H.-L.L.); 2 Laboratory of Fruit Quality Biology, Zhejiang Provincial Key Laboratory of Horticultural Plant Integrative Biology, Zijingang Campus, Zhejiang University, Hangzhou 310058, China; Donald.Grierson@nottingham.ac.uk; 3 Plant Sciences Division, School of Biosciences, Sutton Bonington Campus, University of Nottingham, Loughborough LE12 5RD, UK

**Keywords:** NAC transcription factor, fruit ripening, fruit quality, hormones, texture, color, flavor

## Abstract

The NAC transcription factor (TF) family is one of the largest plant-specific TF families and its members are involved in the regulation of many vital biological processes during plant growth and development. Recent studies have found that NAC TFs play important roles during the ripening of fleshy fruits and the development of quality attributes. This review focuses on the advances in our understanding of the function of NAC TFs in different fruits and their involvement in the biosynthesis and signal transduction of plant hormones, fruit textural changes, color transformation, accumulation of flavor compounds, seed development and fruit senescence. We discuss the theoretical basis and potential regulatory models for NAC TFs action and provide a comprehensive view of their multiple roles in modulating different aspects of fruit ripening and quality.

## 1. Introduction

Fruits originate from the reproductive organ of plants, carry, nourish and protect the developing seeds and generally act as an aid to seed dispersal and propagation of the species. Fleshy fruits are also an important dietary source for humans and animals because they provide a rich variety of nutrients such as carbohydrates, vitamins, antioxidants, trace elements and dietary fiber. Fruit development and ripening is a unique period in the life cycle of higher plants, which usually includes five stages: fertilization of the egg cell, cell division and differentiation, cell expansion, fruit ripening and fruit senescence [1,2]. Fruit ripening occurs after seed development is completed, although parthenocarpic fruits can ripen without seeds.

Fruits can be generally divided into climacteric and non-climacteric types, according to whether there is a respiratory peak during their ripening. At the onset of ripening of climacteric fruit, there is a peak of both respiration and ethylene synthesis, which is necessary for the initiation of ripening. Non-climacteric fruits, however, do not show a significant respiratory peak during ripening, and ethylene production remains at a low level, which mean that ethylene is not a necessary condition for ripening of non-climacteric fruits [3]. Some fruit species, however, have both climacteric and non-climacteric behaviors, depending on the cultivar (melon, blueberry), ripening stage (kiwifruit), and preharvest or postharvest period (raspberry). At the mature stage, when fruit and seed development are completed, fruit usually ripen by undergoing a series of physiological and biochemical changes, such as color transformation, alterations in texture, accumulation of flavor compounds, which are the result of specific spatio-temporal changes in expression of many ripening-related genes. The ripening process is regulated by TFs, which can activate or inhibit the expression of downstream ripening-related target genes by binding to their promoter regions and fine-regulate the fruit ripening process [2]. Many families of TFs have been demonstrated to be involved in the regulation of fruit ripening, such as the MADS-Box (*MCM 1*, *AGAMOUS*, *DEFICIENS*, *SRF-box*) family [4], MYB (v-myb avian myeloblastosis viral oncogene homolog) family [5,6]. The AP2/ERF (APETALA 2/Ethylene response factor) family, acts downstream of the ethylene signaling pathway and initiates the expression of a large number of ripening related genes [7]. Other active TF families include the ARF (Auxin response factor) family, which are key components in auxin signal transduction [8,9], the HD-Zip (Homeodomain leucine zipper) family [10], the SPL (SQUAMOSA promoter binding protein-like) family [11,12], the bHLH (basic helix-loop-helix) family [13,14], and the NAC (NAM, ATAF1, ATAF2 and CUC2) family [15].

The NAC TF family is specific to plants and there are a large number of family members. With the continuous development and improvement of bioinformatic techniques, more and more NAC TFs have been identified in different plant species. A total of 19,997 NAC TFs from 150 species have been included in the plant transcription factor database, of these, 101 are distributed in tomato, 328 in rice, 138 in *Arabidopsis thaliana*, and 280 in tobacco (http://planttfdb.gao-lab.org, accessed on 31 January 2022). *NAM* (*No Apical Meristem*) was the first NAC gene discovered in Petunia in 1996. Petunia embryos carrying the *NAM* mutation were unable to form the shoot apical meristem and showed fused cotyledons, and most died at the seedling stage. A small number of occasional shoots showed abnormal flower organ development [16]. *ATAF1*/*2* (*Arabidopsis Transcription Activator Factors1/2*) and *CUC2* (*Cup-shaped Cotyledon2*) were identified from *Arabidopsis thaliana* in 1997. CUC2 was shown to have sequence homology and functional similarity to *NAM* in Petunia and was found to have partial functional redundancy with CUC1. *CUC2* mutants showed abnormal stem apical meristem development, cup-shaped cotyledons and sepals fused with stamens [17]. *ATAF1*/*2* were found to be involved in stress responses and senescence regulation in plants [18,19]. Since *NAM*, *CUC2* and *ATAF1*/*2* genes all have a conserved amino acid sequence at the N-terminal, this conserved domain was named the NAC domain using the first letter of the names of these three genes and proteins containing NAC domains were named NAC proteins [17]. NAC TFs can be involved in many biological processes regulating plant growth and development, including response to external stress [20], flower organ formation [21], establishment of organ boundaries and plant morphology [22,23], secondary cell wall thickening [24], shoot and root apical meristem formation [16,17], lateral root development [25,26], fiber development [27], senescence regulation [28] and fruit development [15]. In addition, increasing numbers of studies have shown that NAC TFs also play an important regulatory role in fruit ripening [15,29,30]. However, despite their importance in fruits, there is a lack of a systematic review of NAC TFs in the field of fruit ripening. In this paper, we review the NAC TFs that are involved in regulating fruit ripening and quality, focusing on their molecular regulatory mechanisms, and discuss a model outlining the roles that NAC TFs play in regulating fruit ripening and the determination of fruit quality.

## 2. Structure and Subcellular Localization of NAC TFs

**Figure 1:**
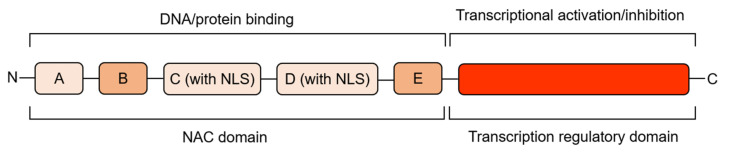
The structure of NAC proteins. The NAC domain at the N-terminal of the NAC protein is divided into five conserved sequence regions: A, B, C, D, and E. The C-terminal is a highly variable transcriptional regulatory domain [31,32,35]. The color depth of each region in the figure represents the degree of variability of this region.

**Figure 2:**
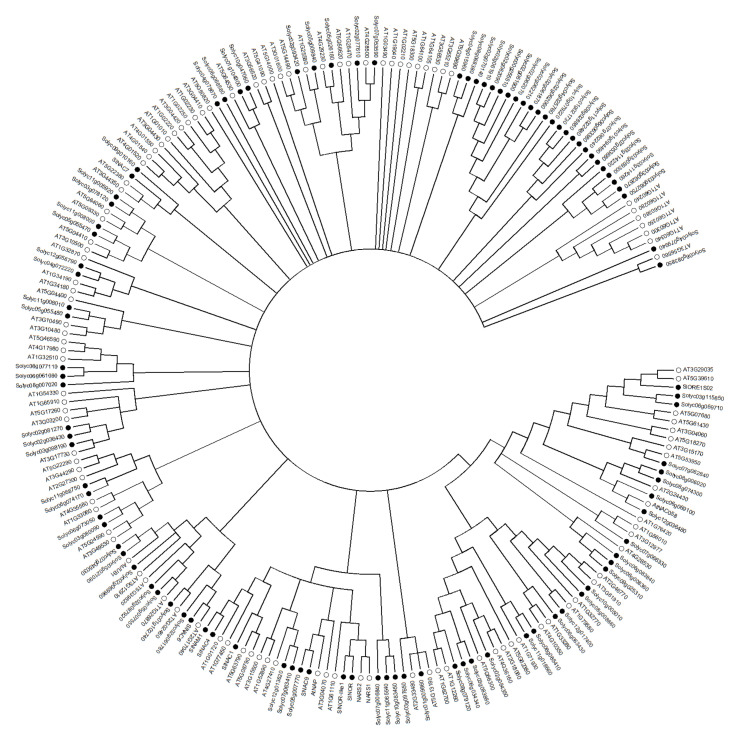
Phylogenetic tree of the NAC family TFs comparing tomato and *Arabidopsis thaliana*. The figure was made using MEGA7.0 software, with the ClustalW method to align sequences, the Neighbor-Joining method to build the tree, and the Bootstrap method to test it (500 times). In this figure, the hollow circles represent the NAC proteins in *Arabidopsis thaliana* and the solid circles represent the NAC proteins in tomato, and the minimum degree of confidence of each branch is 50%.

The N-terminal regions of NAC proteins contain a highly conserved NAC domain, consisting of about 150 amino acids, which can bind DNA and other proteins, and contains five (A, B, C, D, E) conserved sequence regions (Figure 1). Regions A, C, and D are highly conserved in different species, while regions B and E are relatively variable. Regions C and D contain predicted nuclear localization signal (NLS), which may be related to nuclear localization of TFs and identification of specific cis-acting elements of target gene promoter regions, and regions D and E are responsible for physical binding to DNA [31,32]. According to the similarity of the amino acid sequences in their NAC domains, NAC proteins can be divided into two groups, I and II, with 14 subgroups in group I and 4 subgroups in group II [31]. Some conserved basic amino acids in the N-terminal region of NAC protein generate a positive charge-rich surface, which is likely to be directly involved in the binding of NAC proteins to DNA [33], and the core binding sequences favored by NAC proteins have been identified as CGT[G/A] [32], but not all NAC proteins recognize the same sequence, for *Arabidopsis thaliana* CBNAC (Calmodulin-binding NAC protein), GCTT is the core binding sequence [34]. In addition, NAC proteins can act as homodimers or heterodimers, and dimerization is necessary for stable binding to DNA [32]. The C-terminal region of NAC proteins is a transcriptional regulatory domain with strong sequence diversity. Although the C-terminal sequences are highly variable, some common characteristics also exist in this region. There is a high frequency and repetition of certain amino acids, and some serine, threonine, proline, glutamate or acidic residues-rich regions are present. In addition to this, 13 conserved domains can be distinguished. These motifs are distributed in different subgroups of NAC proteins and NAC proteins in the same subgroup contain the same motifs [31,32,35]. Based on the similarity of amino acid sequences, a phylogenetic tree of NAC family TFs comparing tomato (model plant for fruit ripening studies) and model plant *Arabidopsis thaliana* was constructed (Figure 2), to help understand the evolutionary relationship between NAC TFs related to fruit ripening and quality mentioned below. The crystal structure of the NAC domain of ANAC from *Arabidopsis thaliana* was the first determined precise structure of a NAC protein by X-ray crystallography [33]. The results showed that the structure is a symmetrical homodimer, with six antiparallel β-sheet and three α-helices in each monomer. A short antiparallel β-sheet and hydrogen bond/salt bridge between the arginine and glutamate side-chains of the two monomers mediates dimerization. This is different from the classical helix-turn-helix structure and indicates that the NAC domains generate a new transcription factor protein folding structure–an antiparallel β-sheet surrounded by several helix elements [33].

Most of the NAC TFs are localized in the nucleus, but some are localized in the cytoplasm and organelles. In addition, members of a special class, called NAC membrane-bound TFs (or NTM1-like, NTLs), are normally anchored in a membrane (such as plasma membrane, endoplasmic reticulum membrane, or nuclear membrane) in an inactive form. When stimulated by hormones or environmental signals, they are released from the membrane and transferred to the nucleus to regulate the expression of their target genes [36,37,38]. Previous studies have shown that membrane-mediated transcriptional regulation is a common regulatory mechanism [37]. Genome-wide predictive analysis showed that there are 13 membrane-localized NAC TFs in *Arabidopsis thaliana* [39], 5 membrane-localized NAC TFs in rice [35], and 13 membrane-localized NAC TFs in tomato [40]. The possibility that there are other forms of cellular localization of NAC TFs needs further investigation.

## 3. NAC TFs Regulate Fruit Ripening and Development of Quality Attributes

### 3.1. NAC TFs Are Involved in Plant Hormone Biosynthesis and Signal Transduction

Fruit ripening is regulated by various plant hormones, such as ethylene [41,42], abscisic acid (ABA) [43], brassinosteroids (BR) [44], auxins (IAA) [45], gibberellins (GA) [46], jasmonic acid (JA) [47], salicylic acid (SA) [45], melatonin [48], etc. Different plant hormones can jointly regulate fruit ripening through synergistic or antagonistic effects and there are complex mechanisms of interaction between them (“cross-talk”) during fruit ripening [42,49,50]. Ethylene and ABA are two core hormones which play an important role in regulating the ripening process of climacteric and non-climacteric fruits, respectively, although both may participate in climacteric fruit ripening [30,42,51].

The ethylene biosynthesis precursor in plants is methionine (Met), which is transformed into S-adenosyl methionine (SAM) through the methionine cycle. SAM is converted to 1-aminocyclopropane-1-carboxylic acid (ACC) under the action of ACC synthase (ACS), and this is then converted to ethylene by ACC oxidase (ACO). Subsequently, ethylene signals are transmitted to the nucleus through a series of signal transduction elements such as ethylene receptor (ETR), ethylene-insensitive 2 (EIN2), EIN3/EIN3-like (EILs) and ethylene response factors (ERFs), which ultimately initiate the expression of a large number of fruit ripening-related genes [4,41]. In the ABA metabolic pathway, 9-*cis*-epoxycarotenoid dioxygenase (NCED) and ABA 8′-hydroxylase (CYP707A) are key enzymes in ABA biosynthesis and decomposition, respectively. ABA activates downstream targets such as *ABA-responsive element binding proteins* (*AREBs*) and *ABA-responsive element binding factors* (*ABFs*), as well as ethylene biosynthesis through a signaling cascade pathway consisting of the PYR/PYL/RCAR protein family, type 2C protein phosphatase (PP2Cs) and SNF1-related kinase subfamily 2 (SnRK2s), promoting fruit ripening [42,43]. Many studies have shown that NAC TFs can participate in the biosynthesis and signal transduction of plant hormones to regulate the fruit ripening process.

In tomato fruits (*Solanum lycopersicum*), the unripe phenotype of *non-ripening* (*nor*) mutants has long been thought to be caused by the loss of function of the *SlNOR* gene, which was identified as NAC-SlNOR and considered to be a core transcription factor regulating tomato fruit ripening [52] (United States Patent, Patent No.: US 6,762,347 B1). Later, however, Gao et al. [53] and Wang et al. [54] obtained *SlNOR* gene-edited mutants using CRISPR/Cas9 technology and found that the ripening of these gene-edited fruit was only partially inhibited. This was inconsistent with the complete inhibition of fruit ripening phenotype shown by *nor* mutant fruit. The explanation of this major difference was suggested to be that *nor* was a gain-of-function mutant, rather than due to a loss-of-function of *SlNOR*. The original *nor* mutation generated a premature stop codon, leading to the production of residual truncated SlNOR (United States Patent, Patent No.: US 6,762,347 B1). Gao et al. [55] analyzed the function of SlNOR and SlNOR truncated protein (SlNOR186) in fruit ripening of the *nor* mutants and found that SlNOR can positively regulate fruit ripening by binding to the promoter of *SlACS2*, a key gene for ethylene biosynthesis, and activating its expression. SlNOR186 truncated protein, on the other hand, could still bind to the promoters of ripening-related genes but could not activate their normal transcription. In addition, the binding of full length SlNOR protein to the promoters of its target genes was competitively inhibited by the truncated protein SlNOR186. These results confirmed that the *nor* mutant is a gain-of-function mutant, as previously suggested [53,54].

Further work showed that under oxidative stress, SlNOR will undergo a posttranslational modification of sulfoxidation, which weakens the ability of SlNOR to bind to and activate promoters of downstream ripening-related target genes. Met sulfoxide reductases (MSR), E4 and SlMsrB2 were able to partially repair oxidized SlNOR (SlNORox) and restore its DNA-binding ability through interaction with SlNOR. Meanwhile, *E4* and *SlMsrB2* could be transcriptionally activated as downstream target genes of SlNOR [56]. In addition, SlNOR is also a direct regulatory target of RIPENING INHIBITOR (SlRIN, sometimes referred to as SlMADS-RIN) and SlAREB1, a transcription factor downstream of the ABA signaling pathway. SlRIN and SlAREB1 both bind directly to the promoter region of *SlNOR* and activate its expression, and further induce the expression of *SlACS2*, *SlACS4* and *SlACO1* downstream of SlNOR, which enhances ethylene synthesis. This confirms earlier suggestions that the transcriptional regulation of *SlNOR* by SlAREB1 was involved in mediating ABA and ethylene crosstalk during fruit ripening [57,58], but other NAC proteins have also been found to be involved in regulating ripening.

Tomato NAC TF SlNOR-like1 (also referred to as SlNAC3/SNAC4/SlNAC48) has the highest amino acid sequence homology (62.84%) with SlNOR [59,60] and has multiple targets affecting ethylene and ABA concentrations and signaling [60,61,62]. By silencing *SlNOR-like1* with virus-induced gene silencing (VIGS), the ripening process in the silenced part of the fruit was significantly inhibited [60,61,62,63]. Homozygous mutant fruit of *SlNOR-like1* obtained by CRISPR/Cas9 technology showed a 14 day delay in ripening initiation, compared to wild type fruit, and aspects of fruit ripening, such as ethylene biosynthesis, fruit softening, and color transformation were significantly inhibited, and the fruit finally reached an orange-red color [60]. Further studies found that SlNOR-like1 can directly bind to the promoters of key ethylene biosynthesis key genes *SlACS2*, *SlACS4*, *SlACS8* and *SlACO6* and activate their expression [60,62]. SlNOR-like1 can also directly bind to the promoters of *SlCYP707A1* and *SAPK3*, involved in ABA catabolism and signal transduction pathways, activating *SAPK3* and inhibiting *SlCYP707A1*. In addition, it can interact with related proteins SAPK3, SlPYL9, SlACS2 and SlACO1, positively regulating fruit ripening by promoting ethylene biosynthesis and ABA signal transduction [62].

Silencing *SNAC9* (also named *SlNAC19*/*NAC-like*, *activated by Apetala3*/*Pistillata* (*SlNAP2*)) in tomato by VIGS also inhibited the ripening process of the silenced part of fruit [61,62,63], although the fruit softened earlier [63]. Ethylene biosynthesis and signal transduction pathways were inhibited but the expression of *SlNECD1* and *SlNCED2* in the ABA biosynthesis pathway were activated, resulting in the increased accumulation of ABA in fruit [61,63]. It was further found that SNAC9 could interact with key proteins SlPYL9 and SlAREB1 in the ABA signal transduction pathway, indicating that SNAC9 could regulate tomato fruit ripening by simultaneous affecting the biosynthesis and signal transduction pathways of ethylene and ABA [61,62,63].

Ma et al. [64] and Meng et al. [65] tested the effects of overexpression and antisense inhibition of *SlNAC1* (also named *SlNAC033*) in tomato, respectively, and found that *SlNAC1*-overexpressing fruit could not ripen normally and finally became yellow or orange. Ethylene synthesis was significantly inhibited, but the fruit softened earlier [64]. Antisense inhibition, on the other hand, delayed ripening but eventually the fruit became dark red. The ethylene peak was delayed, but the eventual peak value was higher than that of wild-type fruit, and the fruit softening rate slowed down [65]. Further experiments show that SlNAC1 can directly bind to the promoter regions of *SlACS2* and *SlACO1* in the ethylene biosynthesis pathway and inhibit their expression, negatively regulating ethylene biosynthesis [64]. SlNAC1 could also enhance the expression of key ABA biosynthesis genes, *SlNECD1* and *SlNCED2*, and promote the accumulation of ABA in fruit, with multiple effects on fruit ripening [64,65].

SlNAC4 was found to be a positive regulator of tomato fruit ripening. Silencing *SlNAC4* by RNA interference (RNAi) delayed aspects of ripening and carotenoid accumulation was inhibited. The expression levels of the key genes in ethylene biosynthesis, *SlACS2*, *SlACS4*, *SlACO1*, *SlACO3*, and the ethylene response factor *SlERF1*, were significantly inhibited in silenced fruit. In addition, the expression of *SlNAC4* could not be induced by ethylene and SlNAC4 could interact with SlRIN and SlNOR at the protein level. These results suggest that SlNAC4 may be upstream of ethylene biosynthesis by regulating the activities of SlRIN and SlNOR, or alternatively, might regulate fruit ripening through ethylene independent pathways [66].

Jian et al. [67] found that SlNAC6 may induce the early expression of key genes *SlACS2*, *SlACS4* and *SlACO1* in ethylene biosynthesis, and also activate the expression of *SlNCED1*, *SlNCED2*, *SlABA2*, *aldehyde oxidase 1* (*SlAO1*), and *SlAO2* in the ABA biosynthesis pathways, thus promoting ethylene and ABA accumulation, and positively regulating fruit ripening. The expression of *SlNAC7* reached a peak at the color breaking stage during tomato fruit ripening and was induced by a variety of plant hormones such as ethylene and ABA, suggesting that SlNAC7 may be involved in regulating tomato fruit ripening by mediating plant hormone signals [68].

When *JUNGBRUNNEN1* (*AtJUB1*), a known NAC TF in *Arabidopsis thaliana*, was overexpressed in tomato under control of the 35S promoter, the transgenic tomato plants showed dwarfing and developmental changes, and fruit ripening was delayed. Further studies found that AtJUB1 had a similar regulation mode in tomato as in *Arabidopsis thaliana* and could directly bind to the promoter region of *GA 3-oxidase 1* (*GA3ox1*) and *DWARF4* (*DWF4*) in the GA and BR biosynthesis pathways, and *PIF4*, a positive regulator of cell elongation, and inhibited their expression. It could also bind to the promoter region of *DELLA*, a gene encoding a growth inhibitor protein, and activates its expression. Meanwhile, AtJUB1 indirectly inhibited the ethylene biosynthesis and signal transduction related genes *ACS*, *ACO*, *SlERF.H15*, and *SlRIN* to regulate the fruit ripening process [69].

Recently, Gao et al. [70] identified a new NAC TF, SlNAM1, in tomato and obtained homozygous gene-edited mutants of *SlNAM1* using CRISPR/Cas9 technology. It was found that the timing of the ethylene peak and ripening onset in *SlNAM1* mutant fruits were both delayed, whereas they were advanced in *SlNAM1*-overexpressing fruit. However, *SlNAM1*-manipulation had no effect on the peak value of ethylene. Further studies showed that SlNAM1 could directly bind to the promoters of key ethylene biosynthesis genes *SlACS2* and *SlACS4* and activate their expression. The above results indicated that SlNAM1 may redundantly regulate the expression of *SlACS2* and *SlACS4* together with other TFs, and it may affect the start time of enhanced ethylene production (system II onset) through an as yet unknown mechanism, so as to positively regulate the onset of tomato fruit ripening.

**Figure 3:**
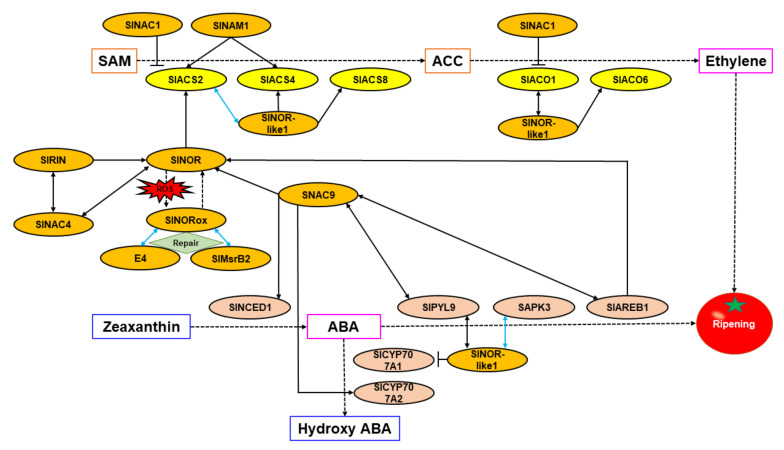
Ethylene and ABA biosynthesis and signal transduction regulated by NAC TFs in tomato. In this figure, black solid one-way arrows represent transcriptional activation, the T symbols represent transcriptional inhibition, black two-way arrows represent protein interaction, and the blue two-way arrows represent the operation of both protein interaction and transcriptional activation.

The transcriptional regulatory networks elucidated for ethylene and ABA biosynthesis and signal transduction mediated by NAC TFs in tomato are shown in Figure 3.

The role of NAC TFs in plant hormone biosynthesis and signal transduction has also been investigated in other fruits. In apples (*Malus domestica*), for example, both MdNAC1 and MdNAC2 can interact with REVERSION-TO-ETHYLENE SENSITIVITY1a (MdRTE1a) and MdRTE1b, important regulators of ethylene signal transduction. *MdNAC1* and *MdRTE1a* are mainly expressed in young fruits, while *MdNAC2* and *MdRTE1b* are highly expressed during fruit ripening. These results suggest that MdNAC1 and MdRTE1a may be involved in regulating the development of young fruits, while MdNAC2 and MdRTE1b play a synergistic role in fruit ripening through regulating ethylene signal transduction [71]. Zhang et al. [72] identified 13 NAC genes differentially expressed during fruit development and ripening in apples. During postharvest storage, accumulation of *MdNAC1a* and *MdNAC78* transcripts was inhibited by ethylene and induced by the ethylene perception inhibitor 1-methylcyclopropene (1-MCP). The transcript levels of *MdNAC2*, *MdNAC26*, *MdNAC41*, *MdNAC57*, *MdNAC80*, *MdNAC91*, *MdNAC119* and *MdNAC141* were consistent with the ethylene production rate, while *MdNAC1*, *MdNAC16* and *MdNAC32* did not respond to 1-MCP. This suggests that some NAC genes may participate in the fruit ripening process in an ethylene-dependent process, while others act in an ethylene-independent manner. When *MdNAC18.1*, a homolog of *SlNOR* in tomato, was overexpressed in the *nor* mutant, the expression of the key ethylene biosynthesis gene *SlACS2* was enhanced, the non-ripening phenotype of *nor* fruit could be partially restored, and *MdNAC18.1* was suggested to be an indicator of apple harvest date, and fruit firmness at harvest and after harvest. Therefore, MdNAC18.1 may control apple fruit ripening in a conserved way, by regulating ethylene production [73].

In papaya fruits (*Carica papaya* L.), CpNAC3 and CpMADS4 directly bind to the promoter regions of *CpERF9* and *CpEIL5*, respectively, key genes in the ethylene signal transduction pathway, and activate their transcription. CpNAC3 could interact with CpMADS4, and the interacting proteins had a stronger transcriptional activation effect on *CpERF9* and *CpEIL5*. These results suggest that CpNAC3 and CpMADS1 can independently or synergistically activate the ethylene signal transduction pathways and positively regulate fruit ripening in papaya [74].

In banana fruits (*Musa acuminata*), Shan et al. [75] identified six NAC TFs named MaNAC1-MaNAC6, that were associated with fruit ripening. These six TFs showed different expression patterns in fruit peels and flesh when fruits were naturally ripened (i.e., relying on endogenous ethylene), or treated with 1-MCP and ethylene alone or in combination. MaNAC1 and MaNAC2 could interact with the ethylene signal transduction element MaEIL5 to participate in banana fruit ripening. In a follow-up study, Shan et al. [76] and Wei et al. [77] further enriched knowledge of the banana fruit ripening regulatory network mediated by MaNAC1 and MaNAC2. It was found that MdNAC1 and MdNAC2 could directly bind to the promoter region of *MaERF11*, a negative regulator of ethylene biosynthesis, and inhibit its transcription, so as to activate the expression of the ethylene biosynthesis genes *MaACS1* and *MaACO1*, the downstream target genes of MaERF11, and promote ethylene biosynthesis. The ethylene response suppressor *RTE1-HOMOLOG 1* (*RTH1*), a negative regulator of fruit ripening, also acts as a downstream target of MaNAC2 and is subject to its transcriptional inhibition. However, RING E3 ligase XA21 Binding Protein3 (MaXB3) could interact with MaNAC2, MaACS1 and MaACO1 and promote their degradation by the ubiquitination pathway, thus inhibiting ethylene biosynthesis and downstream responses at the transcriptional or post-translational level. *MaXB3* is also a downstream target gene of MaNAC1 and MaNAC2 and is subject to their transcriptional inhibition. Transient and ectopic overexpression of MaXB3 in banana fruits and tomato significantly delayed fruit ripening. Therefore, MaNAC1 and MaNAC2 may be involved in regulating banana fruit ripening by mediating the cascade of events promoted by ethylene. Li et al. [78] treated banana fruit with ethylene, and identified 10 NAC TFs by transcriptome analysis, MaNAC009/016/033/083/094/095/129/131/040/074, associated with banana fruit ripening. The expression levels of these 10 NAC TFs were all up-regulated after ethylene treatment, indicating they were directly or indirectly dependent on ethylene. Yan et al. [79] found that the expression levels of *MaNAC42* and *MaMsrB2* were significantly increased in banana fruits under oxidative stress. Further studies showed that MaNAC42 can directly bind to promoters of a series of oxidative stress and fruit ripening related genes, such as *dehydration-responsive element binding 1* (*MaDREB1*) and *MaERF113*, and ectopic overexpression of *MaNAC42* significantly delayed dark-induced leaf senescence in *Arabidopsis thaliana*. The sulfoxylation of MaNAC42 reduced its binding and ability to activate downstream target genes. MaMsrB2 could repair the oxidative damage to MaNAC42 by interacting with MaNAC42, thus restoring the DNA binding ability of MaNAC42. These results suggested that MaNAC42 could negatively regulate the ripening and senescence of banana fruits induced by oxidative stress and that its regulatory capacity was affected by the redox modification mediated by reactive oxygen species (ROS) and MaMsrB2.

In peach fruits (*Prunus persica*), PpNAP1, PpNAP4 and PpNAP6 have been suggested to participate in the regulation of peach fruit ripening by regulating ethylene biosynthesis and responding to ABA signals [80]. Prupe.4G187100, a homolog of tomato SlNOR, may control fruit ripening time and the ripening process in a conservative manner dependent of ethylene [81]. Guo et al. [82] recently identified two TFs by RNA-Seq, *PpNAC.A59* and *PpERF.A16*, that were highly expressed during peach fruit ripening. This study found that PpNAC.A59 could directly bind to the promoter region of *PpERF.A16* and activate its expression. In addition, *PpACS1* and *PpACO1*, key genes for ethylene biosynthesis, and *PpendoPGM*, a key gene for cell wall degradation, could act as downstream target genes of *PpERF.A16* and are transcriptionally activated by it. Both stable genetic transformation and transient transformation of *PpNAC.A59* and *PpERF.A16* in tobacco and in peach fruit showed that PpNAC.A59 promoted ethylene biosynthesis and fruit softening by activating the ERF signaling pathway, and positively regulated fruit ripening.

In kiwifruit (*Actinidia deliciosa*/*Actinidia chinensis*), exogenous methyl jasmonate (MeJA) can synergistically enhance the induction of ripening by exogenous ethylene. This involves the induction of *AdNAC2* and *AdNAC3* and the subsequent increase in endogenous ethylene. AdNAC2 and AdNAC3 can directly bind to the promoter region of *AdACS1* and transactivate it to promote endogenous ethylene production. These results suggest that AdNAC2 and AdNAC3 mediated cross-talk between MeJA and ethylene in the kiwifruit ripening process [83]. AcNAC1, AcNAC2, AcNAC3 and AcNAC4 could directly bind to the promoter region of *AcACS1*, a key gene in the ethylene biosynthesis pathway, and activate its expression. AcNAC2-4 are responsible for regulating ethylene biosynthesis during injury induction and ripening initiation (System I) and fruit ripening (System II) and play a positive regulatory role in kiwifruit ripening [84]. AdNAC6 and AdNAC7 can also directly bind to the promoter regions of *AdACS1* and *AdACO1* and activate their expression to promote ethylene biosynthesis and fruit ripening, but the transcripts of *AdNAC6* and *AdNAC7* could be targeted and degraded by Ade-microRNA164 (miR164). This degradation pathway is located downstream of ethylene signaling and is inhibited by ethylene. Subcellular localization analysis showed that AdNAC6 and AdNAC7 were distributed in both the nucleus and cytoplasm, and AdNAC6 and AdNAC7 could form homodimers or heterodimers localized only in the nucleus. However, the presence of miR164 would prevent AdNAC6 and AdNAC7 from entering the nucleus and cause them to remain in the cytoplasm. Further studies found that the miR164-NAC TFs pathway was conserved in apple, pear, peach, strawberry, citrus, grape and banana fruits [85]. In addition, ethylene inducible AdNAC2, AdNAC72 and AdMsrB1 could positively regulate ethylene production and ripening of kiwifruit under oxidative stress. AdMsrB1 could reduce dabsyl-Met-R-sulfoxide (Met-R-SO) induced by oxidative stress to regenerate the ethylene precursor Met, while AdNAC2 and AdNAC72 could directly bind to the promoter of *AdMsrB1* and activate its expression, thus expanding the Met pool, and AdNAC2 could trans-activate *AdACS1* and increase the content of ACC, the direct precursor of ethylene, and then promote ethylene production and fruit ripening [86].

In strawberry fruits (*Fragaria* × *ananassa*/*Fragaria chiloensis*), Moyano et al. [87] identified six NAC TFs, FaNAC006, FaNAC021, FaNAC022, FaNAC035, FaNAC042 and FaNAC092, related to fruit development and ripening through genome-wide and microarray transcriptomic analysis. Of these, FaNAC035 (also named Ripening Inducing Factor (FaRIF)) promotes the accumulation of ABA by inducing the expression of *FaNCED3* and *zeaxanthin epoxidase* (*FaZEP*), two key genes in the ABA biosynthesis pathway. FaNAC035 also participates in ABA signal transduction by regulating the expression patterns of *FaHVA22*, *FaSnRK2.6*, *ELONGATED HYPOCOTYL5* (*FaHY5*) and *B-box-containing protein 19* (*FaBBX19*) and is therefore involved in cross-talk between various plant hormones, positively regulating fruit ripening. There is also a feedback mechanism whereby the ABA level promotes *FaNAC035* expression, which in turn can increase ABA concentration [88]. Carrasco-Orellana et al. [89] cloned *FcNAC1* from strawberry and showed that the transcriptional abundance of *FcNAC1* increased during strawberry fruit ripening. *In silico* analysis showed that the promoter sequence of *FcNAC1* contained cis-response elements for a variety of hormones, such as ABA and auxin. These results suggested that FcNAC1 could participate in regulating fruit ripening by responding to upstream hormone signals.

In citrus fruits (*Citrus reticulata*), Zhu et al. [90] systematically analyzed a citrus ABA-deficient mutant using multiomics and cytology and found that ABA played a positive role in promoting citrus fruit ripening, and the ABA-deficient mutant was caused by the abnormally high expression of *CrNAC036*. Further studies showed that CrNAC036 could bind to the promoter region of *CrNCED5*, a key gene for ABA biosynthesis, and inhibit its expression, while CrMYB68 could interact with CrNAC036 to enhance the transcriptional inhibition of *CrNCED5*, synergistically inhibiting ABA accumulation and the process of citrus fruits ripening.

In melon fruits (*Cucumis melo* L.), *MELO3C016540* (*CmNAC-NOR*) is most closely related to *SlNAC-NOR*, a positive regulator of tomato fruit ripening. Non-synonymous mutations of *CmNAC-NOR* lead to significant delays in the initiation of melon fruit ethylene biosynthesis and ripening. When *CmNAC-NOR* was introduced into non-climacteric melon varieties, ethylene biosynthesis was enhanced and the climacteric ripening ability of fruit was partially restored, suggesting that CmNAC-NOR plays an important role in the climacteric ripening process of melon fruits [91].

In monocotyledon oil palm fruits (*Elaeis guineensis*), ethylene biosynthesis and cell wall degradation pathways are significantly activated during fruit ripening, when NAC TFs are actively expressed. *EgNAC6* and *EgNAC7*, two genes with homology to *SlNOR* in tomato, are strongly induced by ethylene. This suggested that fruit ripening in monocotyledons and dicotyledons may be regulated by a conserved molecular program in which NAC TFs may play an important role [92]. It has been suggested previously that there are at least three modes of ethylene regulation, all involving NAC genes in slightly different roles in different types of fruits from monocotyledons and dicotlyledons [53].

In pear fruits (*Pyrus ussuriensis*), the expression levels of *PuNAC2* and *PuNAC8* (inhibited by 1-MCP) were significantly up-regulated after the respiratory peak and may be involved in ethylene biosynthesis and signal transduction during fruit ripening, while the expression of *PuNAC21* was significantly down-regulated, suggesting that it may be a negative ripening regulator [93].

### 3.2. NAC TFs and Fruit Textural Changes

The texture changes of fruit are an important feature of fruit ripening. They are due mainly to changes in cell wall structure, which causes fruit softening but can also include lignification [94]. Fruit softening is the most common texture change during ripening and is almost always caused by changes in cell wall composition and structure, including pectin degradation, depolymerization of cellulose and hemicellulose, relaxation of cellulose-hemicellulose network, changes caused by cell wall expansion, etc. [95,96,97,98]. These cell wall changes are caused by a series of enzymes related to cell wall degradation, including polygalacturonase (PG), pectin methylesterase (PME), pectate lyase (PL), β-galactosidase (TBG), xyloglucan endotransglycosylase (XTH), endoglucanase (CEL) and expansins (EXP), etc. [97,99,100,101,102]. Fruit lignification is mainly manifested by the accumulation of lignin in the cell wall, which increases fruit hardness. This is a relatively rare texture change during fruit ripening and has a negative impact on fruit quality. The lignification of the cell wall is mainly due to the activation of key enzymes in the lignin biosynthesis pathway, including phenylalanine ammonia lyase (PAL), 4-coumarate: coenzyme a ligase (4CL), cinnamyl alcohol dehydrogenase (CAD), peroxidases (PODs), etc. [94]. Current studies have found that there are NAC binding sites in the promoter regions of some genes related to fruit softening and lignification pathways, and NAC TFs can regulate the expression level of these genes by binding to their promoters, thus regulating the texture change during fruit ripening.

In tomato fruits (*Solanum lycopersicum*), SlNOR could promote cell wall degradation by directly binding to the promoter region of cell wall degradation-related gene *SlPL* and activating its expression [55]. SlNOR-like1 could target and activate *SlPG2a*, *SlPL*, *SlCEL2* and *SlEXP1*, and interact with SlPG2 to promote tomato fruit softening [60,103]. SNAC9 negatively regulates ABA biosynthesis and also inhibits the expression of cell wall degradation-related genes *SlEXP*, *SlPG*, *SlPL*, and also *lipoxygenase* (*SlLOX*), thus slowing down the fruit softening process [63], while SlNAC1 could promote ABA accumulation in fruits, which induces and increases the transcript levels of *SlPG*, *SlEXP1*, *SlCEL1* and *acid invertase 1* (*SlWiv1*), and promotes fruit softening [64,65]. Gao et al. [104] constructed CRISPR-Cas9-mediated homozygous mutants of *SlNAC4* and *SlNAC4*-overexpressing plants and found that the softening process of *SlNAC4* gene-edited fruits was significantly inhibited, while that of *SlNAC4*-overexpressing fruits was accelerated. Further studies found that, SlNAC4 could directly bind to the promoter regions of key cell wall degradation genes *SlEXP1* and *SlCEL2* and activate their expression, thereby positively promoting fruit softening.

In kiwifruit (*Actinidia deliciosa*), AdNAC6 and AdNAC7 could directly bind to the promoter region of the cell wall degradation-related gene *endo-β-mannanase* (*AdMAN1*) and activate its expression, thus promoting the softening process during kiwifruit ripening [85].

In strawberry fruits (*Fragaria* × *ananassa*/*Fragaria chiloensis*), the *FcNAC1* promoter contains secondary wall NAC binding elements, which can be bound by NAC TFs associated with cell wall biosynthesis, and FcNAC1 activates transcription from the promoter of *FcPL*. This suggests that FcNAC1 could respond to upstream cell wall biosynthesis related NAC TFs and participates in downstream pectin metabolism to promote strawberry fruit softening [89]. FaNAC035 can positively regulate the expression of cell wall degradation genes *β-xylosidase 3* (*FaXYL3*), *FaPL2*, *FaPL3*, *FaPL4*, *endo-1*,*4-β-glucanase 15* (*FaGH9B15*, encodes a CEL), *ARABIDOPSIS DEHISCENCE ZONE POLYGALACTURONASE 2* (*FaADPG2*), *FaEXP1*, *FaEXP2*, *FaEXP3*, and *FaPME39*, as well as regulating branch fluxes from the phenylalanine pathway to limit lignin biosynthesis and promote fruit softening [88].

In loquat fruits (*Eriobotrya japonica*), EjNAC1 has 59.55% identity with VASCULAR-RELATED NAC-DOMAIN 7 (VND7) in *Arabidopsis thaliana*, a master regulator of secondary cell wall formation. The expression level of *EjNAC1* was closely related to the degree of fruit lignification after harvest. Further analysis showed that EjNAC1 could transcriptionally activate *EjPAL1* and *Ej4CL1*, which encode two key enzymes in the lignin biosynthesis pathway. Transient overexpression of *EjNAC1* in tobacco leaves induced the expression of endogenous lignin biosynthesis genes and accumulation of lignin, suggesting that EjNAC1 promotes lignification of loquat fruits by activating lignin biosynthesis-related genes [105]. Another NAC TF, *EjNAC3*, whose transcriptional level was also consistent with the degree of fruit lignification, can physically bind to the promoter of the *EjCAD-like* gene and trans-activate its transcription, indicating EjNAC3 is an activator of loquat lignification [106].

In jujube fruits (*Ziziphus jujuba* Mill.), Zhang et al. [107] identified a NAC TF, LOC107435239, that was highly related to pericarp lignification of winter jujube. Through heterologous expression in *Arabidopsis thaliana* and transient expression in winter jujube pericarp, it was confirmed that LOC107435239 could positively regulate the accumulation of pericarp lignin during winter jujube fruit pigmentation by promoting the expression of *ferulate 5-hydroxylase* (*F5H*). In addition, *ZjNAC13*, *ZjNAC14*, *ZjNAC38* and *ZjNAC41* were highly expressed in half-red and full-red jujube fruit and may be involved in the jujube fruit ripening process [108].

Based on the known information of NAC TFs in fruit texture regulation, we can predict a common mechanism of NAC TFs in regulating fruit texture. For one thing, NAC TFs can directly target genes encoding enzymes that catalyze changes related to degradation of cell wall components (mainly pectin and cellulose) or the lignification pathway. For another, NAC TFs can indirectly affect fruit texture by regulating endogenous ABA and ethylene levels, which promote the expression of downstream genes that bring about cell wall modification.

### 3.3. NAC TFs and Fruit Color Transformation

Fruit ripening is often accompanied by an obvious color transformation, which is caused by dynamic changes in various pigment levels, involving chlorophyll degradation and carotenoid and anthocyanin accumulation [2,42,109,110]. Color transformation is controlled by key enzymes in different pigments metabolic pathways, such as STAY-GREEN (SGR) and pheophorbide a oxygenase (PAO) in the chlorophyll degradation pathway [109], deoxyxylulose 5-phosphatesynthase (DXS) and phytoene synthase (PSY), phytoene desaturase (PDS), lycopene-cyclase (LCY) in the carotenoid biosynthesis pathway [111], and chalcone synthase (CHS), chalcone isomerase (CHI), flavanone 3-hydroxylase (F3H), and dihydroflavonol 4-reductase (DFR), anthocyanidin synthase (ANS), uridine diphosphate-glucose: flavonoid 3-O-glucosyltransferase (UTFG) in the anthocyanins biosynthesis pathway [112]. It has been found that NAC TFs directly target different genes in these metabolic pathways and regulate color transformation during fruit ripening.

In tomato fruits (*Solanum lycopersicum*), SlNOR activates *geranylgeranyl pyrophosphate synthase 2* (*SlGgpps2*) in the carotenoid biosynthesis pathway and promotes the accumulation of carotenoids during fruit ripening [55]. SlNOR-like1 also directly binds to the promoter region of *SlGgpps2* and *SlSGR1* in the carotenoid biosynthesis and chlorophyll degradation pathways and activates their expression to promote color transformation during fruit ripening [60]. SlNAC1 targets the promoter region of *SlPSY1* in the carotenoid biosynthesis pathway and inhibits its expression, thus inhibiting carotenoid accumulation during fruit ripening [64,65], while SlNAC4 positively regulates the transcript level of *SlPSY1* and promotes carotenoid accumulation during fruit ripening [66].

In apple fruits (*Malus domestica*), the transcript level of *MdNAC52* increases continuously during the coloring process of apple fruit. Further studies showed that MdNAC52 could interact with the promoters of positive regulatory factors *MdMYB9*, *MdMYB11* and the key enzyme *leucoanthocyanidin reductase* (*LAR*) in the proanthocyanidins (PA) and anthocyanidin biosynthesis pathways and activate their expression to promote the accumulation of PA and anthocyanidins during fruit ripening. Moreover, it could act as a downstream target gene of MdHY5 in response to light signals [113]. Zhang et al. [114] identified a NAC TF MdNAC42 in red-fleshed apples, whose transcription abundance was positively correlated with anthocyanin accumulation during fruit ripening. The flavonoid biosynthesis pathway was activated in apple callus overexpressing *MdNAC42*, and anthocyanin content was significantly increased. Moreover, MdNAC42 could interact with MdMYB10, an important positive regulator of anthocyanin biosynthesis, to promote anthocyanin accumulation during apple fruit ripening. The transcript level of *MdNAC9* is positively correlated with the accumulation of flavonol pigments that accumulate in apple fruits. In callus overexpressing *MdNAC9*, the transcript level of *flavonol synthase* (*MdFLS*), a key gene in the flavonol biosynthesis pathway, and flavonol content were significantly increased. Further experimental evidence showed MdNAC9 positively regulates the accumulation of flavonols during apple fruit ripening by binding to the promoter of *MdFLS* and activating its expression [115].

In papaya fruits (*Carica papaya* L.), the expressions of *CpNAC1* and the key carotenoid biosynthesis genes *CpPDSs* were up-regulated during natural and propylene-induced ripening and their transcript levels were positively correlated with the accumulation of carotenoids. CpNAC1 directly binds to the promoter regions of *CpPDS2* and *CpPDS4* and activates their expression, thus positively regulating the accumulation of carotenoids during papaya fruit ripening [116]. CpNAC2 could interact with the promoters of the key carotenoid biosynthesis genes *CpPDS2*, *CpPDS4*, *ξ*-*carotene desaturase* (*CpZDS*), *CpLCY-e* and *carotene hydroxylase-b* (*CpCHY-b*) and activate their transcription. The transcript level of ethylene signal transduction element *CpEIN3a* was positively correlated with the accumulation of carotenoids during fruit ripening and could also target *CpPDS4* and *CpCHY-b* to activate their transcription. Moreover, *CpEIN3a* could interact with CpNAC2, which significantly enhanced the transactivation of *CpPDS2*, *CpPDS4*, *CpLCY-e* and *CpCHY-b* by CpNAC2. These investigations showed that CpNAC2 and CpEIN3a could activate the carotenoid biosynthesis pathway alone or synergistically to positively regulate carotenoid accumulation during papaya fruit ripening [117].

Peach fruit (*Prunus persica*) can be either red-fleshed, yellow fleshed, or white. In peach fruits, the expression levels of NAC TF BLOOD (BL) and PpMYB10.1, an important positive regulator of anthocyanin biosynthesis, were significantly increased in blood-fleshed fruit during the late development stage compared with the transcript levels in non-red-fleshed fruit. Further studies showed that BL could form a heterodimer with PpNAC1 (ppa008301m) to further activate the transcription of *PpMYB10.1*, and PpMYB10.1 could promote downstream anthocyanin accumulation by interacting with bHLH protein and activating the expression of *PpDFR* and *PpUFGT*, key genes in the anthocyanin biosynthesis pathway. The trans activation activity of BL-PpNAC1 heterodimer in early fruit development was inhibited by SQUAMOSA promoter-binding-like 1 (PpSPL1), thus restricting anthocyanin accumulation in early fruit development [118]. PpNAC1 is also widely believed to be an important TF regulating fruit ripening in date [119,120,121,122]. In addition, PpNAC19 could inhibit the promoter activity of *carotenoid cleavage dioxygenase 4* (*PpCCD4*), a key gene for carotenoid degradation, to promote carotenoid accumulation during fruit ripening [123].

Comparative transcriptome analysis between red- and white-fleshed strawberry fruits (*Fragaria* × *ananassa*) at the white and full red stage revealed that members of the NAC family may be involved in regulating anthocyanin accumulation during strawberry fruit ripening [124]. For example, FaNAC035 could induce anthocyanin accumulation during fruit ripening by promoting ABA accumulation [88].

In kumquat fruits (*Fortunella crassifolia* Swingle), Gong et al. [125] found that the process of fruit degreening and coloration based on carotenoid accumulation was significantly accelerated by red light, and by analyzing transcriptome data, showed that NAC TF FcrNAC22 transcript level was significantly induced under red light. The manipulation of *FcrNAC22* in the tomato and citrus callus stable expression systems and transient expression in citrus fruit showed that FcrNAC22 has a positive regulatory effect on fruit coloration. FcrNAC22 could bind to the promoters of key genes in the carotenoid metabolism pathway, including *FcrLCYB1*, *β-carotene hydroxylase 2* (*FcrBCH2*) and *FcrNCED5*, and activate their expression, indicating that FcrNAC22 promotes citrus fruit coloration by activating the carotenoid metabolic pathway.

In litchi fruits (*Litchi chinensis*), transcripts of *LcNAC13* increased with fruit ripening and anthocyanin accumulation. Further studies showed that LcNAC13 could directly target the promoter regions of the anthocyanin biosynthesis-related genes *LcCHS1*, *LcCHS2*, *LcCHI*, *LcF3H*, *LcF3′H*, *LcDFR* and *LcMYB1*, and inhibit their expression. *LcR1MYB1* was also highly expressed during fruit ripening, moreover it can interact with LcNAC13 and weaken or even reverse the transcriptional inhibition of LcNAC13 on its target genes. This suggests that LcNAC13 and LcR1MYB1 may antagonistically regulate anthocyanin accumulation during litchi fruit ripening [126].

In pear fruits (*Pyrus pyrifolia*), Ahmad et al. [127] identified 185 NAC TFs within the genome. Through RNA-Seq and real-time quantitative PCR (RT-qPCR) analysis, PpNAC61/70/172/176/23 were predicted to be involved in regulating blue light-induced fruit coloration, while PpNAC56 may be related to fruit ripening.

### 3.4. NAC TFs and Accumulation of Fruit Flavor Compounds

The accumulation of sugars, organic acids and volatile aroma compounds is an important event in the fruit ripening process, which determines the final flavor and nutritional quality of fruit. Sugars in fruits mainly come from photosynthates imported from vegetative organs, although some are also derived from fruit chloroplasts photosynthesis prior to ripening. The sugars mainly accumulate in the form of starch during the immature stage of fruit development, and this is gradually transformed into soluble sugars such as maltose, sucrose, glucose and fructose through the starch degradation pathway and sugar metabolic network during fruit ripening. Malic acid and citric acid are the most common organic acids in fruits. Unlike sugars, organic acids are usually synthesized in situ using precursors such as free sugars, metabolites of starch and cell walls. Organic acids can enter the tricarboxylic acid cycle as substrates for respiration and are also linked with glucose metabolism, and content are influenced by several factors [42,128,129]. In addition, many volatile aroma compounds are formed during fruit ripening, which can be divided into aldehydes, esters, alcohols, furans, ketones, terpenes, acids, lactones and sulfur compounds. However, usually only a few volatile compounds are responsible for the overall aroma and unique taste of specific fruits [130,131,132]. Volatile aroma compounds are mainly synthesized from pathways related to metabolism of fatty acids, carotenoids, branched amino acids and phenylalanine derivatives [130,131,132]. NAC TFs can regulate the formation of flavor compounds during fruit ripening, which affects fruit ripening and sensory quality.

In tomato fruits (*Solanum lycopersicum*), SNAC9 binds to the promoter regions of *SENESCENCE-ASSOCIATED GENE113* (*SlSAG113*), *SlSGR1* and *SlPAO* genes, related to senescence and chlorophyll degradation, and *SlNCED1*, *SlCYP707A2* and *ATP-binding cassette transporter G family member 40* (*SlABCG40*) genes related to ABA metabolism and transport, and activates their expression. In addition, *SlNOR* is a direct downstream target of SNAC9, and SlNOR could further activate transcription from ripening- and senescence-related genes *SlABCG40*, *SlERT1B*, *SlSAG113*, *SlSGR1*, *SlSAG15*, *YELLOW LEAF SPECIFIC4* (*SlYSL4*), pheophytinase (*SlPPH*), and *kelch-repeat F-box protein targeting type-B Arabidopsis Response Regulator 20* (*SlKFB20*), to control the photosynthetic cycle of leaves by promoting leaf senescence, thus regulating fruit yield and sugar accumulation during ripening [133,134]. ORESARA1 S02 (SlORE1S02) responds to a variety of senescence-inducible stimuli and interacts with TFs GOLDEN2-LIKE 1 (SlGLK1) and SlGLK2, associated with chloroplast maintenance, and serves as a target of post-transcriptional regulation by miR164. Silencing *SlORE1S02* in tomato using RNAi technology increased the expression level of *SlGLK2* and decreased the expression level of senescence related gene *SlSAG12*. The leaves showed delayed aging and enhanced carbon assimilation, and the fruit quantity, starch content and soluble solid accumulation were increased. These results suggest that SlORE1S02 could control fruit yield and sugar accumulation by positively regulating leaf senescence, limiting the time available for leaf photosynthesis and the production and transportation of photosynthates [135].

In banana fruits (*Musa acuminata*), the expression levels of *MaNAC67-like*, *EIN3 binding F-box-1* (*MaEBF1*) and a series of starch degradation related genes were significantly inhibited at low temperatures. As a result, the fruit could not ripen normally, and the starch content was significantly increased. Further studies showed that MANAC67-like could bind to the promoter regions of *β-amylase* (*MaBAM6*), *starch excess* (*MaSEX4*) and *maltose transporter* (*MaMEX1*), key genes for starch degradation, and activate their expression. Moreover, MaNAC67-like could interact with ethylene signal transduction TF MaEBF1 to further enhance the capacity for transcriptional activation to *MaBAM6* and *MaSEX4*. These results suggest that the inhibition of expression of *MaNAC67-like* and its interacting proteins is one of the main ways that low temperature inhibits banana fruit ripening [136].

In watermelon fruits (*Citrullus lanatus*), *ClNAC92*/*54*/*29*/*71*/*16*/*74*/*72*/*02b*/*21*/*01*/*40a*/*32*/*75a*/*02a*/*05*/*28*/*57*/*43*/*56a*/*79b*/*87*/*68*/*53b* were expressed both in the pulp and the vascular tissue and may be related to fruit ripening and quality [137]. Recently, Wang et al. [138] found that *ClNAC68* was expressed at a high level in fruit flesh. By using CRISPR/Cas9 technology to knock out *ClNAC68* in watermelon, it was found that the soluble solids content and sucrose accumulation of *ClNAC68* knockout mutant fruit were significantly reduced. Further studies revealed that ClNAC68 positively regulates sucrose accumulation during fruit ripening by directly binding to the promoter region of *invertase* (*ClINV*) and inhibiting its expression.

In citrus fruits (*Citrus reticulata* Blanco), CitNAC62 and CitWRKY1 could transactivate the promoter of a citric acid degradation related gene *aconitase 3* (*CitAco3*), thus promoting citric acid degradation during citrus fruit development and ripening. Moreover, it was found that there was an interaction between CitNAC62 and CitWRKY1 in the nuclear region, which appeared to increase the transcriptional activation of *CitAco3*. However, subcellular localization showed that CitWRKY1 was located in the nucleus, while CitNAC62 was mainly located in the cytoplasm, indicating that the interaction between CitNAC62 and CitWRKY1 may depend on the movement of CitNAC62 in the cell [139].

In persimmon fruits (*Diospyros kaki*), 95% CO_2_ treatment induces anaerobic respiration, which effectively reduces the content of soluble tannins in postharvest persimmon fruit to achieve fruit de-astringency, due to the precipitation of the tannins by the increase in acetaldehyde that results from the induction of anaerobic respiration. NAC TFs *DkNAC1*, *DkNAC2*, *DkNAC3*, *DkNAC5* and *DkNAC6* respond strongly to CO_2_ treatment and transcript levels of all except *DkNAC2*, showed a high correlation with the degree of de-astringency in persimmon fruit. The expression of *DkNAC2* lagged behind the process of fruit de-astringency and may be related to the process of fruit ripening and senescence [140]. Jin et al. [141] found that the expression level of *DkNAC7* was consistent with the degree of fruit de-astringency under high concentration CO_2_ treatment, and DkNAC7 could regulate the process of postharvest persimmon fruit de-astringency by trans activating the fruit de-astringency regulator *DkERF9* and related gene *pyruvate decarboxylase 2* (*DkPDC2*). Jamil et al. [142] isolated two other NAC TFs, DkNAC13 and DkNAC16 from persimmon fruits and their transcript abundance was consistent with the degree of de-astringency of persimmon fruits mediated by high concentration of CO_2_. DkNAC13 and DkNAC16 could activate the promoter regions of de-astringency related genes *DkERF9* and *alcohol dehydrogenase 1* (*DkADH1*), respectively, thereby activating the de-astringency process of persimmon fruit.

In kiwifruit (*Actinidia arguta/Actinidia chinensis/Actinidia deliciosa*), the level of accumulation *of Actinidia arguta* terpenoids is significantly higher than that of *Actinidia chinensis*. This is mainly due to the high expression level of *terpene synthase1* (*AaTPS1*) in *Actinidia arguta* at the ripening stage. Further studies showed that *AaTPS1* is a direct downstream target activated by NAC TFs AaNAC2, AaNAC3 and AaNAC4. However, in *Actinidia chinensis* AcNAC2, AcNAC3 and AcNAC4 could not perform normal transcriptional activation due to mutations in the NAC binding site in the promoter region of *AcTPS1*, which resulted in limited expression of *AcTPS1* and biosynthesis of terpenoids [143]. In addition, AdNAC6 and AdNAC7 could also interact with the promoter region of *AaTPS1* and activate its expression, thereby promoting the synthesis of terpene aroma compounds during kiwifruit ripening [85].

In peach fruits (*Prunus persica*), PpNAC1 promotes the biosynthesis of volatile esters during fruit ripening by directly binding to the promoter region of alkyl transferase *alcohol acyltransferase 1* (*PpAAT1*) and activating its expression. This pathway is downstream of ethylene signal and conserved in tomato (SlNOR-*SlAAT1*) and apple fruit (MdNAC5-*MdAAT1*) [144].

In strawberry fruits (*Fragaria × ananassa*), FaNAC035 positively regulates the expressions of *SUCROSE PHOSPHATE SYNTHASE1* (*FaSPS1*), *SUCROSE SYNTHASE1* (*FaSUS*), *EMISSION OF BENZENOIDS II* (*FaEOBII*), *DOF-like* (*FaDOF2*), *EUGENOL SYNTHASE2* (*FaEGS2*) and *NEROLIDOL SYNTHASE1* (*FaNES1*) to promote the accumulation of sucrose and volatile compounds (phenylpropanoid eugenol, linalool and nerolidol) during fruit ripening [88].

### 3.5. NAC TFs and Seed Development

Seed development is an important part of fruit ripening, especially for fruits with seeds as the main edible part, such as the caryopsis of species of Poaceae or Gramineae. Many studies have shown that seed quality is closely related to fruit ripening stage [145,146,147,148]. In recent years, NAC TFs have also been reported to play an important regulatory role in seed development during fruit development and ripening.

In tomato fruits (*Solanum lycopersicum*), SlNOR-like1 plays an important role in the regulation of seed development. *SlNOR-like1* gene-edited mutants have abnormal seed development, with significantly reduced seed number and weight, and extremely low germination rates [60]. This is consistent with the seed phenotype of *SlNOR-like1*-RNAi transgenic lines obtained by Han et al. [149] with reduced seed size, loss of embryo sac, collapse of endosperm, reduced pollen viability, and inhibition of the JA biosynthesis pathway in flowers. Kou et al. [63] also reported that seeds from *SlNOR-like1* silenced fruit obtained by VIGS had similar phenotypes.

In watermelon fruits (*Citrullus lanatus*), the germination of the seeds of *ClNAC68* gene-edited fruit was inhibited, seedlings were stunted and the contents of free IAA in seeds and roots were significantly reduced. Further studies showed that, ClNAC68 could positively regulates the accumulation of IAA during fruit ripening and promotes normal seed development by directly binding to the promoter region of the *IAA-amino synthetase* (*ClGH3.6*) (IAA deactivator) in the IAA signaling pathway and inhibiting its expression [138].

In *Arabidopsis thaliana* siliques, NAC-REGULATED SEED MORPHOLOGY 1 (NARS1) (also named NAC2) and NARS2 (also named NAM) are involved in regulating the development of embryo and integument in *Arabidopsis thaliana* seeds. Knockout of *NARS1* or *NARS2* alone did not affect normal seed development, while simultaneous knockout of *NARS1* and *NARS2* led to seed development defects. This suggested that NARS1 and NARS2 have functional redundancy in regulating *Arabidopsis thaliana* seed development [150].

In rice caryopses (*Oryza sativa*), three rice NAC TFs ONAC020, ONAC023 and ONAC026 are specifically expressed at high levels during caryopsis development and ripening, and both ONACO20 and ONACO23 could interact with ONAC026. These three TFs participated in the regulation of caryopsis size and weight through transcriptional activation or transcriptional inhibition of downstream genes [151]. *ONAC127* and *ONAC129* were also specifically expressed in rice caryopses, mainly in pericarp, and played an important role in maintaining caryopsis filling under heat stress. Further studies showed that sugar transporter genes *sugar will eventually be exported transporter 4* (*OsSWEET4*), *monosaccharide transporter 6* (*OsMST6*) and abiotic stress response genes *ERF protein associated with tillering and panicle branching* (*OsEATB*) and *multi-stress-responsive gene 2* (*OsMSR2*) were the downstream target genes of ONAC127 and ONAC129. ONAC127 and ONAC129 could inhibit transcription of both *OsSWEET4* and *OsEATB*, while ONAC127 played a transcriptional activation role in *OsMST6* and *OsMSR2*. Moreover, ONAC127 and ONAC129 could form heterodimers, in which, ONAC129 negatively regulated the transcriptional activation capacity of ONAC127. These results suggested that ONAC127 and ONAC129 could synergically regulate the caryopsis filling process by acting on sugar transport and abiotic stress responses [152].

In grapefruits (*Vitis vinifera*), *VvNAC26* was highly expressed in ovules of seedless grape varieties, while *VvMADS9* had the same expression pattern as *VvNAC26* and could interact with VvNAC26 at the protein level. The heterologous overexpression of *VvNAC26* in tomato induced the expression of genes related to the ethylene and abscisic acid biosynthesis pathways, which resulted in earlier fruit ripening, deeper coloration, smaller fruit size and abnormal seed development, which suggested that VvNAC26 may promote fruit ripening and senescence and regulate seed development by activating ethylene and ABA biosynthesis [153].

In soybean (*Glycine max* (L.) Merr.), *GmNAC1*/*2*/*3*/*4*/*5*/*6* were differentially expressed at different stages of seed filling, suggesting that these NAC TFs may be related to pod ripening [154].

In apricot fruits (*Prunus armeniaca* L./*Prunus sibirica*), *NAC* gene was predicted to be associated with fruit ripening date [155]. Xu et al. [156] identified a total of 102 NAC TFs within the genome of Siberian apricot. The expression of these NAC TFs was analyzed by RNA-seq and RT qPCR, and it was found that *PsNAC6*/*13*/*46*/*51*/*41*/*67*/*37*/*59* were highly expressed at the fruit ripening stage. It was concluded that they might participate in the process of Siberian apricot fruit ripening, whereas PsNAC6/13/51/41/67 might be involved in kernel development and ripening.

### 3.6. NAC TFs and Fruit Senescence

Fruit ripening and senescence are inseparable, and the latter follows on from the former. At the late stage of fruit ripening, fruits begin to enter the senescence stage under oxidative stress of ROS [157]. Current studies have found that many NAC TFs are also involved in fruit senescence regulation.

In strawberry fruits (*Fragaria* × *ananassa*), miRNA high-throughput sequencing and degradome group analysis associated with the senescence process during ambient post-harvest storage found that *NAC domain containing protein 87*, *NAC domain containing protein 38* and *NAC domain transcriptional regulator superfamily protein* are important targets of the miR164 family members, including mdm-miRNA164d-1ss21AC, mdm-miRNA164e, ptc-miR164f-1ss21TA, and might promote postharvest senescence of strawberry fruits through positive regulation of cell senescence and death [158]. Compared with ambient temperature storage, low temperature storage at 4 °C delayed the senescence of strawberry fruits and induced increased expression levels of mdm-miRNA164d-1ss21AC and mdm-miRNA164e, while the expression levels of *NAC domain containing protein 38* and *NAC domain transcriptional regulator superfamily* protein were significantly inhibited [159]. Wang et al. [160] also confirmed by SRNAome and transcriptome analysis that ripening related NAC TFs *NAC domain-containing protein 100-like*, *NAC domain-containing protein 100-like isoform 1* and *NAC domain-containing protein 21/22-like* were direct targets of miR164a, miR164b, and miR164c. These results suggested that the miR164 family could inhibit postharvest senescence processes and maintain postharvest quality of strawberry fruits by targeted degradation of the transcripts of ripening and senescence related NAC TFs.

In citrus fruits (*Citrus sinensis* Osbeck), CitNAC has high homology with TFs AtNAP and PvNAP, which are related to plant organ senescence. *CitNAC* is only expressed in ripening or senescence stage of fruit organs, suggesting that it might be involved in regulating these processes [161].

In litchi fruits (*Litchi chinensis*), the expression of *LcNAC1* was associated with senescence signals and exogenous senescence stress factors. The expression of *LcNAC1* in fruits in which senescence was delayed by low temperature was significantly higher than that in naturally senescing fruits, suggesting that LcNAC1 may negatively regulate senescence. The TF LcMYC2 could act upstream of *LcNAC1* and positively regulate its expression, while LcNAC1 could activate the transcription of its downstream target gene *alternative oxidase a* (*LcAOX1a*, related to reactive oxygen species regulation and energy metabolism), but the expression of *LcAOX1a* was inhibited by LcWRKY1, an interacting protein of LcNAC1. These results indicated that LcNAC1 and LcWRKY1 regulate the progression of fruit senescence by respectively activating or repressing *LcAOX1a* [162]. In addition, Jiang et al. [163] found that calmodulin 1 (LcCAM1) interacted with senescence-related TFs LcNAC13 and LcWRKY1, but under oxidative stress, methionine oxidation of LcCAM1 would enhance the binding ability of LcNAC13 and LcWRKY1 to downstream target genes, while LcMsrA1 and LcMsrB1 could interact with LcCAM1 to repair the oxidative damage of LcCAM1. Thus, LcMsrA1/B1-LcCAM1-LcNAC13/LcWRKY1 constitute an interaction network regulating fruit senescence.

In *Arabidopsis thaliana* siliques, the senescence of *NARS1* and *NARS2* double mutants was delayed, suggesting that NARS1 and NARS2 play a positive regulatory role in the senescence of siliques [150]. AtNAP, another NAC TF, has also been implicated in the senescence of *Arabidopsis thaliana* siliques. When *AtNAP* is knocked out, the senescence of *Arabidopsis thaliana* siliques was delayed by 4–5 days. Based on the pattern of ethylene release and respiration intensity of siliques in response to exogenous hormones, it is speculated that AtNAP might mediate ABA induced stomatal closure, thereby providing sufficient oxygen for ethylene synthesis and rapid respiration of siliques, positively regulating siliques senescence in *Arabidopsis thaliana* [164]. In addition, Mizzotti et al. [165] identified a NAC transcription factor AtNAC058 by transcriptomics that may be involved in the maturation and senescence of *Arabidopsis thaliana* siliques. In the *AtNAC058* knockout mutant, chlorophyll degradation of siliques was accelerated and senescence was advanced. Therefore, AtNAC058 may be a negative regulator of silique maturation and senescence.

All NAC TFs mentioned in this review are summarized in Table 1. In different plant species, there are similarities and differences in the mechanism of NAC TFs regulating fruit ripening. For example, NAC TFs can participate in the biosynthesis and signal transduction of ethylene and ABA to regulate climacteric fruit ripening, while mainly participate in the biosynthesis and signal transduction of ABA to regulate non-climacteric fruit ripening. However, the hormone response mediated by NAC TFs (with ethylene and ABA as the core hormones) might be a common mechanism to regulate the ripening of different fruit species. In addition, NAC TFs can directly regulate various metabolic pathways related to fruit ripening, such as fruit softening, color transformation, flavor compounds accumulation, etc. In different fruit species, the metabolic pathways targeted by NAC TFs show both conservation and specificity, which is probably due to differences in the recruitment of NAC TFs to the regulatory circuits during evolution of different plant species.

## 4. Conclusions and Prospects

**Figure 4:**
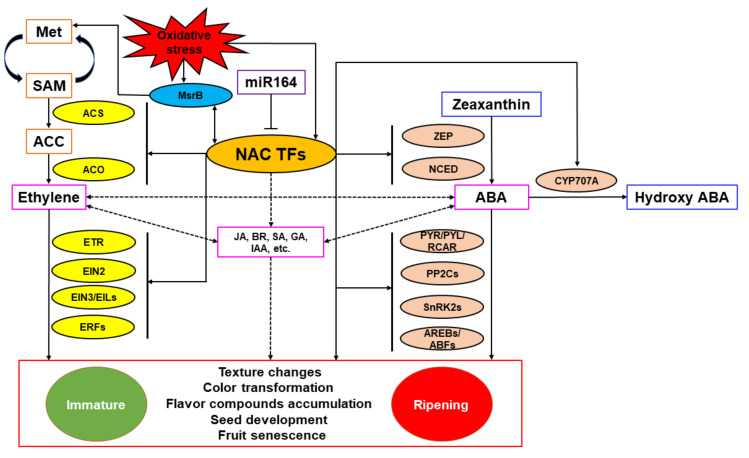
Model of NAC TFs regulating fruit ripening and quality formation. On the one hand, NAC TFs regulate fruit ripening and quality formation by regulating hormone biosynthesis and signal transduction. On the other hand, NAC TFs can directly regulate fruit metabolic pathways determining fruit quality attributes, such as texture change, color transformation and flavor compounds accumulation. The transcripts of NAC TFs can be degraded by miR164.

NAC TFs regulate fruit ripening mainly by directly activating or inhibiting key genes in the ethylene and ABA biosynthesis and signal transduction pathways or interacting with proteins related to the above pathways. For climacteric fruits, NAC TFs can affect fruit ripening through both ethylene and ABA pathways, while for non-climacteric fruits, NAC TFs mainly affect fruit ripening through the ABA pathway. TFs induced by the action of these hormones upregulate genes required to catalyze essential ripening processes. Meanwhile, NAC TFs can also directly interact with promoter regions of genes encoding enzymes related to the generation of different aspects of fruit ripening quality (such as color, flavor, texture) or key enzymes and regulatory proteins that regulate fruit quality during fruit ripening. In addition, NAC TFs can synergistically regulate fruit ripening through the MsrB-mediated oxidative stress system, while members of the miR164 family can negatively regulate the transcription abundance of NAC TFs at the post-transcriptional level, antagonizing its regulation of fruit ripening. A model of how NAC TFs regulate fruit ripening and quality formation is shown as Figure 4. It is important to keep in mind that NAC proteins act in concert with other TFs, including RIN, ethylene related and ABA-related TFs, and others, and participate in a network of factors that integrate and regulate different aspects of the ripening process in response to endogenous and environmental signals. Thus, the regulation of any specific ripening gene may involve the participation of several different TFs.

Fruits have important nutritional and economic value, and NAC TFs play a crucial regulatory role in fruit ripening and quality formation. In future research we can envisage the use of NAC TFs as gene manipulation targets to construct fruit varieties with improved ripening quality by transgenic technology. Moreover, other unknown NAC TFs related to fruit ripening are expected to be identified and their roles in determining quality attributes in various plant species clarified. Additional research must be done to identify their downstream target genes and interacting proteins and understand their regulatory mechanisms and interacting partners. This will enrich our knowledge and understanding of the roles of NAC TFs and how they contribute to the wider regulatory network of fruit ripening and quality formation.

## Figures and Tables

**Table 1:** NAC TFs related to fruit ripening and quality formation.

TFs	Fruit Species	Subcellular Localization	Target Gene	Interacting Proteins	Function	Reference
SlNOR	Tomato	Nucleus	*SlACS2*, *SlGgpps2*, *SlPL*, *SlAAT1*, *E4*, *SlMsrB2*, *SlABCG40*, *SlERT1B*, *SlSAG113*, *SlSGR1*, *SlSAG15*, *SlYSL4*, *SlPPH*, *SlKFB20*	SlNAC4, E4, SlMsrB2	Ethylene biosynthesis, carotenoid biosynthesis, fruit softening, volatile ester formation	[53,54,55,56,66,134,144]
SlNOR-like1/SlNAC3/SNAC4/SlNAC48	Tomato	Nucleus	*SlACS2*, *SlACS4*, *SlACS8*, *SlACO6*, *SAPK3*, *SlCYP707A1*, *SlGgpps2*, *SlSGR1*, *SlPG2a*, *SlPL*, *SlCEL2*, *SlEXP1*	SlPG, SlACS2, SlACO1, SAPK3, SlPYL9	Ethylene biosynthesis, ABA signal transduction, carotenoid biosynthesis, chlorophyll degradation, fruit softening, seed development	[59,60,61,62,63,103,149]
SNAC9/SlNAC19/SlNAP2	Tomato	Nucleus	*SlSAG113*, *SlSGR1*, *SlPAO*, *SlNCED1*, *SlABCG40*, *SlCYP707A2*, *SlNOR*	SlPYL9, SlAREB1	Ethylene biosynthesis, ABA biosynthesis and signal transduction, carotenoid biosynthesis, chlorophyll degradation, fruit softening, sugar accumulation	[59,61,62,63,133,134]
SlNAC1/SlNAC033	Tomato	/	*SlACS2*, *SlACO1*, *SlPSY1*	/	Ethylene biosynthesis, ABA biosynthesis, carotenoid biosynthesis, fruit softening	[64,65]
SlNAC4	Tomato	Nucleus	*SlEXP1*, *SlCEL2*	SlRIN, SlNOR	Ethylene biosynthesis and signal transduction, carotenoid biosynthesis, chlorophyll degradation, fruit softening	[66,104]
SlNAC6	Tomato	Nucleus	/	/	Ethylene and ABA biosynthesis	[67]
SlNAC7	Tomato	/	/	/	Ethylene and ABA signal transduction	[68]
SlORE1S02	Tomato	Nucleus	/	SlGLK1, SlGLK2	Sugar accumulation	[135]
AtJUB1	Tomato	Nucleus and cytoplasm	*GA3ox1*, *DWF4*, *PIF4*, *DELLA*	/	Biosynthesis of gibberellin and brassinosteroid	[69]
SlNAM1	Tomato	Nucleus	*SlACS2*, *SlACS4*	/	Ethylene biosynthesis	[70]
MdNAC2	Apple	/	/	MdRTE1a, MdRTE1b	Ethylene signal transduction	[71]
MdNAC1a, MdNAC78, MdNAC2, MdNAC26, MdNAC41, MdNAC57, MdNAC80, MdNAC91, MdNAC119, MdNAC141, MdNAC1, MdNAC16, MdNAC32	Apple	/	/	/	Fruit ripening (predicted)	[72]
MdNAC52	Apple	Nucleus	*MdMYB9*, *MdMYB11*, *LAR*	/	Anthocyanin biosynthesis	[113]
MdNAC42	Apple	Nucleus	/	MdMYB10	Anthocyanin biosynthesis	[114]
MdNAC9	Apple	Nucleus	*MdFLS*	/	Flavonol biosynthesis	[115]
MdNAC5	Apple	/	*MdAAT1*	/	Volatile ester formation	[144]
MdNAC18.1	Apple	/	/	/	Carotenoid biosynthesis, chlorophyll degradation, fruit softening	[73]
CpNAC1	Papaya	Nucleus	*CpPDS2*, *CpPDS4*	/	Carotenoid biosynthesis	[116]
CpNAC2	Papaya	Nucleus	*CpPDS2*, *CpPDS4*, *CpZDS*, *CpLCY-e*, *CpCHY-b*	CpEIN3a	Carotenoid biosynthesis	[117]
CpNAC3	Papaya	Nucleus	*CpERF9*, *CpEIL5*	CpMADS4	Ethylene signal transduction	[74]
MaNAC1	Banana	Nucleus	*MaERF11*, *MaXB3*	MaEIL5	Ethylene signal transduction	[75,76]
MaNAC2	Banana	Nucleus	*MaERF11*, *MaXB3*, *MaRTH1*	MaEIL5, MaXB3	Ethylene biosynthesis and signal transduction	[75,76,77]
MaNAC3, MaNAC4, MaNAC5	Banana	Nucleus	/	/	Fruit ripening (predicted)	[75]
MaNAC6	Banana	Entire cell	/	/	Fruit ripening (predicted)	[75]
MaNAC67-like	Banana	Nucleus	*MaBAM6*, *MaSEX4*, *MaMEX1*	MaEBF1	Starch degradation, ethylene signal transduction	[136]
MaNAC009, MaNAC016, MaNAC033, MaNAC040, MaNAC074, MaNAC083, MaNAC094, MaNAC095, MaNAC129, MaNAC131	Banana	Nucleus	/	/	Fruit ripening (predicted)	[78]
MaNAC42	Banana	Nucleus	*MaDREB1*, *MaERF113*	MaMsrB2	Oxidative stress response, ethylene signal transduction	[79]
PpNAC1 (ppa008301m)	Peach	Nucleus	*PpAAT1*	/	Maturity date, anthocyanin biosynthesis, volatile ester formation	[118,119,120,121,122,144]
BL	Peach	/	*PpMYB10.1*	PpNAC1	Anthocyanin biosynthesis	[118]
PpNAP1, PpNAP4, PpNAP6	Peach	/	/	/	Ethylene biosynthesis and ABA signal transduction (predicted)	[80]
PpNAC19	Peach	Nucleus (predicted)	*PpCCD4*	/	Carotenoid biosynthesis	[123]
Prupe.4G187100	Peach	/	*/*	/	Fruit developmental timing and ripening	[81]
PpNAC.A59	Peach	/	*PpERF.A16*	/	Ethylene biosynthesis and signal transduction, fruit softening	[82]
AaNAC2, AaNAC3, AaNAC4	Kiwifruit	/	*AaTPS1*	/	Terpene synthesis	[143]
AcNAC1, AcNAC2, AcNAC3, AcNAC4	Kiwifruit	/	*AcACS1*	/	Ethylene biosynthesis	[84]
AdNAC2	Kiwifruit	Nucleus, cytoplasm and cell membrane	*AdACS1*, *AdMsrB1*	/	Ethylene biosynthesis	[83,86]
AdNAC3	Kiwifruit	/	*AdACS1*	/	Ethylene biosynthesis	[83]
AdNAC6, AdNAC7	Kiwifruit	Nucleus and cytoplasm	*AdACS1*, *AdACO1*, *AdMAN1*, *AaTPS1*	AdNAC6, AdNAC7	Ethylene biosynthesis, fruit softening, terpene synthesis	[85]
AdNAC72	Kiwifruit	Nucleus	*AdMsrB1*	/	Ethylene biosynthesis	[86]
DkNAC1, DkNAC3, DkNAC5, DkNAC6	Persimmon	/	/	/	Fruit de-astringency	[140]
DkNAC2	Persimmon	/	/	/	Fruit senescence	[140]
DkNAC7	Persimmon	Nucleus	*DkERF9*, *DkPDC2*	/	Fruit de-astringency	[141]
DkNAC13	Persimmon	/	*DkERF9*	/	Fruit de-astringency	[142]
DkNAC16	Persimmon	/	*DkADH1*	/	Fruit de-astringency	[142]
ClNAC92, ClNAC54, ClNAC29, ClNAC71, ClNAC16, ClNAC74, ClNAC72, ClNAC02b, ClNAC21, ClNAC01, ClNAC40a, ClNAC32, ClNAC75a, ClNAC02a, ClNAC05, ClNAC28, ClNAC57, ClNAC43, ClNAC56a, ClNAC79b, ClNAC87, ClNAC53b	Watermelon	Nucleus, chloroplasts, or cytoplasm (predicted)	/	/	Fruit ripening and quality (predicted)	[137]
ClNAC68	Watermelon	Nucleus	*ClINV*, *ClGH3.6*	/	Sugar accumulation, seed development, IAA signal transduction	[138]
NAC domain containing protein 87, NAC domain containing protein 38, NAC domain transcriptional regulator superfamily protein	Strawberry	/	/	/	Fruit senescence	[158,159]
NAC domain-containing protein 100-like, NAC domain-containing protein 100-like isoform 1, NAC domain-containing protein 21/22-like	Strawberry	/	/	/	Fruit senescence	[160]
FaNAC022, FaNAC042	Strawberry	/	/	/	Fruit softening (predicted)	[87]
FaNAC006, FaNAC092	Strawberry	/	/	/	Fruit senescence (predicted)	[87]
FaNAC021	Strawberry	/	/	/	Water balance and/or stress (predicted)	[87]
FaNAC035/FaRIF	Strawberry	/	/	/	ABA biosynthesis and signal transduction, anthocyanin biosynthesis, fruit softening, accumulation of sugars and volatile compounds, energy metabolism	[88]
FcNAC1	Strawberry	Nucleus	*FcPL*	/	Response to hormone signal, fruit softening	[89]
CitNAC	Citrus	/	/	/	Fruit ripening and senescence (predicted)	[161]
CitNAC62	Citrus	Nucleus and cytoplasm	*CitAco3*	CitWRKY1	Citric acid degradation	[139]
CrNAC036	Citrus	Nucleus	*CrNCED5*	CrMYB68	ABA biosynthesis	[90]
FcrNAC22	Kumquat	Nucleus	*FcrLCYB1*, *FcrBCH2*, *FcrNCED5*	/	Carotenoid biosynthesis	[125]
LcNAC1	Litchi	Nucleus, cytoplasm and cell membrane	*LcAOX1a*	LcWRKY1	Regulation of reactive oxygen species and energy metabolism, fruit senescence	[162]
LcNAC13	Litchi	Nucleus	*LcCHS1*, *LcCHS2*, *LcCHI*, *LcF3H*, *LcF3′H*, *LcDFR*, *LcMYB1*, *LcCAM1*	LcR1MYB1	Anthocyanin biosynthesis	[126,163]
NARS1/NAC2	Arabidopsis	Nucleus	/	/	Seed development, fruit senescence	[150]
NARS2/NAM	Arabidopsis	Nucleus	/	/	Seed development, fruit senescence	[150]
AtNAP	Arabidopsis	Nucleus	/	/	Fruit senescence	[164,166]
AtNAC058	Arabidopsis	/	/	/	Chlorophyll degradation, fruit senescence	[165]
ONAC020	Rice	Endoplasmic reticulum	/	ONAC026	Seed development	[151]
ONAC023	Rice	Cytoplasm	/	ONAC026	Seed development	[151]
ONAC026	Rice	Nucleus	/	ONAC020, ONAC023	Seed development	[151]
ONAC127	Rice	Nucleus	*OsSWEET4*, *OsMST6*, *OsEATB*, *OsMSR2*	ONAC129	Sugar transport, abiotic stress response	[152]
ONAC129	Rice	Nucleus	*OsSWEET4*, *OsMST6*, *OsEATB*, *OsMSR2*	ONAC127	Sugar transport, abiotic stress response	[152]
EjNAC1	Loquat	/	*EjPAL1*, *Ej4CL1*	/	Fruit lignification	[105]
EjNAC3	Loquat	Nucleus	*EjCAD-like*	/	Fruit lignification	[106]
CmNAC-NOR	Melon	/	/	/	Ethylene biosynthesis	[91]
EgNAC6, EgNAC7	Oil palm	/	/	/	Fruit ripening (predicted)	[92]
PuNAC2, PuNAC8	Pear	/	/	/	Ethylene biosynthesis and signal transduction (predicted)	[93]
PuNAC21	Pear	/	/	/	Negative regulatory factor of fruit ripening (predicted)	[93]
PpNAC61, PpNAC70, PpNAC172, PpNAC176, PpNAC23	Pear	/	/	/	Fruit coloration induced by blue light (predicted)	[127]
PpNAC56	Pear	/	/	/	Fruit ripening (predicted)	[127]
VvNAC26	Grape	Nucleus	/	VvMADS9	Ethylene biosynthesis, ABA biosynthesis, seed development	[153]
NAC	Apricot	/	/	/	Ripening date	[155]
PsNAC6, PsNAC13, PsNAC46, PsNAC51, PsNAC41, PsNAC67, PsNAC37, PsNAC59	Apricot	Nucleus or chloroplast (predicted)	/	/	Fruit and kernel ripening (predicted)	[156]
LOC107435239	Winter jujube	/	/	/	Lignin accumulation	[107]
ZjNAC13, ZjNAC14, ZjNAC38, ZjNAC41	Chinese jujube	/	/	/	Fruit ripening (predicted)	[108]
GmNAC1, GmNAC2, GmNAC3, GmNAC4, GmNAC5, GmNAC6	Soybean	/	/	/	Seed development	[154]

## References

[1] Klee H.J., Giovannoni J.J. (2011). Genetics and control of tomato fruit ripening and quality attributes. Annu. Rev. Genet..

[2] Giovannoni J., Nguyen C., Ampofo B., Zhong S.L., Fei Z.J. (2017). The epigenome and transcriptional dynamics of fruit ripening. Annu. Rev. Genet..

[3] Alexander L., Grierson D. (2002). Ethylene biosynthesis and action in tomato: A model for climacteric fruit ripening. J. Exp. Bot..

[4] Li S., Chen K.S., Grierson D. (2019). A critical evaluation of the role of ethylene and MADS transcription factors in the network controlling fleshy fruit ripening. N. Phytol..

[5] Fan Z.Q., Ba L.J., Shan W., Xiao Y.Y., Lu W.J., Kuang J.F., Chen J.Y. (2018). A banana R2R3-MYB transcription factor MaMYB3 is involved in fruit ripening through modulation of starch degradation by repressing starch degradation-related genes and *MabHLH6*. Plant J..

[6] Cao H.H., Chen J., Yue M., Xu C., Jian W., Liu Y.D., Song B.Q., Gao Y.Q., Cheng Y.L., Li Z.G. (2020). Tomato transcriptional repressor MYB70 directly regulates ethylene-dependent fruit ripening. Plant J..

[7] Xie X.L., Yin X.R., Chen K.S. (2016). Roles of APETALA2/ethylene-response factors in regulation of fruit quality. Crit. Rev. Plant Sci..

[8] Zhang T., Li W.J., Xie R.X., Xu L., Zhou Y., Li H.L., Yuan C.C., Zheng X.L., Xiao L.T., Liu K.D. (2020). CpARF2 and CpEIL1 interact to mediate auxin-ethylene interaction and regulate fruit ripening in papaya. Plant J..

[9] Khaksar G., Sirikantaramas S. (2020). Auxin response factor 2A is part of the regulatory network mediating fruit ripening through auxin-ethylene crosstalk in Durian. Front. Plant Sci..

[10] Meng L.H., Fan Z.Q., Zhang Q., Wang C.C., Gao Y., Deng Y.K., Zhu B.Z., Zhu H.L., Chen J.Y., Shan W. (2020). *BEL1-LIKE HOMEODOMAIN 11* regulates chloroplast development and chlorophyll synthesis in tomato fruit. Plant J..

[11] Zhu L.S., Liang S.M., Chen L.L., Wu C.J., Wei W., Shan W., Chen J.Y., Lu W.J., Su X.G., Kuang J.F. (2020). Banana MaSPL16 Modulates carotenoid biosynthesis during fruit ripening through activating the transcription of lycopene beta-cyclase genes. J. Agric. Food Chem..

[12] Lai T.F., Wang X.H., Ye B.S., Jin M.F., Chen W.W., Wang Y., Zhou Y.Y., Blank A.M., Gu M., Zhang P.C. (2020). Molecular and functional characterization of the SBP-box transcription factor SPL-CNR in tomato fruit ripening and cell death. J. Exp. Bot..

[13] Song C.B., Shan W., Kuang J.F., Chen J.Y., Lu W.J. (2020). The basic helix-loop-helix transcription factor MabHLH7 positively regulates cell wall-modifying-related genes during banana fruit ripening. Postharvest Biol. Technol..

[14] Zhang L.C., Kang J., Xie Q.L., Gong J., Shen H., Chen Y.A., Chen G.P., Hu Z.L. (2020). The basic helix-loop-helix transcription factor bHLH95 affects fruit ripening and multiple metabolisms in tomato. J. Exp. Bot..

[15] Forlani S., Mizzotti C., Masiero S. (2021). The NAC side of the fruit: Tuning of fruit development and maturation. BMC Plant Biol..

[16] Souer E., van Houwelingen A., Kloos D., Mol J., Koes R. (1996). The *no apical meristem* gene of Petunia is required for pattern formation in embryos and flowers and is expressed at meristem and primordia boundaries. Cell.

[17] Aida M., Ishida T., Fukaki H., Fujisawa H., Tasaka M. (1997). Genes involved in organ separation in Arabidopsis: An analysis of the cup-shaped cotyledon mutant. Plant Cell.

[18] Puranik S., Sahu P.P., Srivastava P.S., Prasad M. (2012). NAC proteins: Regulation and role in stress tolerance. Trends Plant Sci..

[19] Garapati P., Xue G.P., Munné-Bosch S., Balazadeh S. (2015). Transcription factor ATAF1 in arabidopsis promotes senescence by direct regulation of key chloroplast maintenance and senescence transcriptional cascades. Plant Physiol..

[20] Nuruzzaman M., Sharoni A.M., Kikuchi S. (2013). Roles of NAC transcription factors in the regulation of biotic and abiotic stress responses in plants. Front. Microbiol..

[21] Sablowski R.W.M., Meyerowitz E.M. (1998). A homolog of *NO APICAL MERISTEM* is an immediate target of the floral homeotic genes *APETALA3/PISTILLATA*. Cell.

[22] Berger Y., Harpaz-Saad S., Brand A., Melnik H., Sirding N., Alvarez J.P., Zinder M., Samach A., Eshed Y., Ori N. (2009). The NAC-domain transcription factor GOBLET specifies leaflet boundaries in compound tomato leaves. Development.

[23] Kato H., Motomura T., Komeda Y., Saito T., Kato A. (2010). Overexpression of the NAC transcription factor family gene *ANAC036* results in a dwarf phenotype in *Arabidopsis thaliana*. J. Plant Physiol..

[24] Nakano Y., Yamaguchiz M., Endo H., Rejab N.A., Ohtani M. (2015). NAC-MYB-based transcriptional regulation of secondary cell wall biosynthesis in land plants. Front. Plant Sci..

[25] Xie Q., Frugis G., Colgan D., Chua N.H. (2000). *Arabidopsis* NAC1 transduces auxin signal downstream of TIR1 to promote lateral root development. Genes Dev..

[26] He X.J., Mu R.L., Cao W.H., Zhang Z.G., Zhang J.S., Chen S.Y. (2005). AtNAC2, a transcription factor downstream of ethylene and auxin signaling pathways, is involved in salt stress response and lateral root development. Plant J..

[27] Ko J.H., Yang S.H., Park A.H., Lerouxel O., Han K.H. (2007). ANAC012, a member of the plant-specific NAC transcription factor family, negatively regulates xylary fiber development in *Arabidopsis thaliana*. Plant J..

[28] Podzimska-Sroka D., O’Shea C., Gregersen P.L., Skriver K. (2015). NAC transcription factors in senescence: From molecular structure to function in crops. Plants.

[29] Mathew I.E., Agarwal P. (2018). May the fittest protein evolve: Favoring the plant-specific origin and expansion of NAC transcription factors. BioEssays.

[30] Kou X.H., Zhou J.Q., Wu C.E., Yang S., Liu Y.F., Chai L.P., Xue Z.H. (2021). The interplay between ABA/ethylene and NAC TFs in tomato fruit ripening: A review. Plant Mol. Biol..

[31] Ooka H., Satoh K., Doi K., Nagata T., Otomo Y., Murakami K., Matsubara K., Osato N., Kawai J., Carninci P. (2003). Comprehensive analysis of NAC family genes in *Oryza sativa* and *Arabidopsis thaliana*. DNA Res..

[32] Olsen A.N., Ernst H.A., Lo Leggio L., Skriver K. (2005). DNA-binding specificity and molecular functions of NAC transcription factors. Plant Sci..

[33] Ernst H.A., Olsen A.N., Larsen S., Lo Leggio L. (2004). Structure of the conserved domain of ANAC, a member of the NAC family of transcription factors. EMBO Rep..

[34] Kim H.S., Park B.O., Yoo J.H., Jung M.S., Lee S.M., Han H.J., Kim K.E., Kim S.H., Lim C.O., Yun D.J. (2007). Identification of a calmodulin-binding NAC protein as a transcriptional repressor in *Arabidopsis*. J. Biol. Chem..

[35] Olsen A.N., Ernst H.A., Lo Leggio L., Skriver K. (2005). NAC transcription factors: Structurally distinct, functionally diverse. Trends Plant Sci..

[36] Kim Y.S., Kim S.G., Park J.E., Park H.Y., Lim M.H., Chua N.H., Park C.M. (2006). A membrane-bound NAC transcription factor regulates cell division in *Arabidopsis*. Plant Cell.

[37] Seo P.J., Kim S.G., Park C.M. (2008). Membrane-bound transcription factors in plants. Trends Plant Sci..

[38] Wang D.X., Yu Y.C., Liu Z.H., Li S., Wang Z.L., Xiang F.N. (2016). Membrane-bound NAC transcription factors in maize and their contribution to the oxidative stress response. Plant Sci..

[39] Kim S.Y., Kim S.G., Kim Y.S., Seo P.J., Bae M., Yoon H.K., Park C.M. (2007). Exploring membrane-associated NAC transcription factors in *Arabidopsis*: Implications for membrane biology in genome regulation. Nucleic Acids Res..

[40] Bhattacharjee P., Das R., Mandal A., Kundu P. (2017). Functional characterization of tomato membrane-bound NAC transcription factors. Plant Mol. Biol..

[41] Liu M.C., Pirrello J., Chervin C., Roustan J.P., Bouzayen M. (2015). Ethylene control of fruit ripening: Revisiting the complex network of transcriptional regulation. Plant Physiol..

[42] Li S., Chen K.S., Grierson D. (2021). Molecular and hormonal mechanisms regulating fleshy fruit ripening. Cells.

[43] Leng P., Yuan B., Guo Y.D. (2014). The role of abscisic acid in fruit ripening and responses to abiotic stress. J. Exp. Bot..

[44] Baghel M., Nagaraja A., Srivastav M., Meena N.K., Kumar M.S., Kumar A., Sharma R.R. (2019). Pleiotropic influences of brassinosteroids on fruit crops: A review. Plant Growth Regul..

[45] Perez-Llorca M., Munoz P., Muller M., Munne-Bosch S. (2019). Biosynthesis, metabolism and function of auxin, salicylic acid and melatonin in climacteric and non-climacteric fruits. Front. Plant Sci..

[46] Alferez F., de Carvalho D.U., Boakye D. (2021). Interplay between abscisic acid and gibberellins, as related to ethylene and sugars, in regulating maturation of non-climacteric fruit. Int. J. Mol. Sci..

[47] Pena-Cortes H., Barrios P., Dorta F., Polanco V., Sanchez C., Sanchez E., Ramirez I. (2004). Involvement of jasmonic acid and derivatives in plant responses to pathogens and insects and in fruit ripening. J. Plant Growth Regul..

[48] Arnao M.B., Hernandez-Ruiz J. (2020). Melatonin in flowering, fruit set and fruit ripening. Plant Reprod..

[49] Kumar R., Khurana A., Sharma A.K. (2014). Role of plant hormones and their interplay in development and ripening of fleshy fruits. J. Exp. Bot..

[50] Kou X.H., Feng Y., Yuan S., Zhao X.Y., Wu C., Wang C., Xue Z.H. (2021). Different regulatory mechanisms of plant hormones in the ripening of climacteric and non-climacteric fruits: A review. Plant Mol. Biol..

[51] Fenn M.A., Giovannoni J.J. (2021). Phytohormones in fruit development and maturation. Plant J..

[52] Giovannoni J.J. (2004). Genetic regulation of fruit development and ripening. Plant Cell.

[53] Gao Y., Zhu N., Zhu X.F., Wu M., Jiang C.Z., Grierson D., Luo Y.B., Shen W., Zhong S.L., Fu D.Q. (2019). Diversity and redundancy of the ripening regulatory networks revealed by the fruitENCODE and the new CRISPR/Cas9 *CNR* and *NOR* mutants. Hortic. Res..

[54] Wang R.F., Tavano E.C.D.R., Lammers M., Martinelli A.P., Angenent G.C., de Maagd R.A. (2019). Re-evaluation of transcription factor function in tomato fruit development and ripening with CRISPR/Cas9-mutagenesis. Sci. Rep..

[55] Gao Y., Wei W., Fan Z.Q., Zhao X.D., Zhang Y.P., Jing Y., Zhu B.Z., Zhu H.L., Shan W., Chen J.Y. (2020). Re-evaluation of the *nor* mutation and the role of the NAC-NOR transcription factor in tomato fruit ripening. J. Exp. Bot..

[56] Jiang G.X., Zeng J., Li Z.W., Song Y.B., Yan H.L., He J.X., Jiang Y.M., Duan X.W. (2020). Redox regulation of the NOR transcription factor is involved in the regulation of fruit ripening in tomato. Plant Physiol..

[57] Fujisawa M., Nakano T., Shima Y., Ito Y. (2013). A large-scale identification of direct targets of the tomato MADS Box transcription factor RIPENING INHIBITOR reveals the regulation of fruit ripening. Plant Cell.

[58] Mou W.S., Li D.D., Luo Z.S., Li L., Mao L.C., Ying T.J. (2018). SlAREB1 transcriptional activation of *NOR* is involved in abscisic acid-modulated ethylene biosynthesis during tomato fruit ripening. Plant Sci..

[59] Kou X.H., Wang S., Wu M.S., Guo R.Z., Xue Z.H., Meng N., Tao X.M., Chen M.M., Zhang Y.F. (2014). Molecular characterization and expression analysis of NAC family transcription factors in tomato. Plant Mol. Biol. Rep..

[60] Gao Y., Wei W., Zhao X.D., Tan X.L., Fan Z.Q., Zhang Y.P., Jing Y., Meng L.H., Zhu B.Z., Zhu H.L. (2018). A NAC transcription factor, NOR-like1, is a new positive regulator of tomato fruit ripening. Hortic. Res..

[61] Kou X.H., Liu C., Han L.H., Wang S., Xue Z.H. (2016). NAC transcription factors play an important role in ethylene biosynthesis, reception and signaling of tomato fruit ripening. Mol. Genet. Genomics..

[62] Yang S., Zhou J.Q., Watkins C.B., Wu C.E., Feng Y.C., Zhao X.Y., Xue Z.H., Kou X.H. (2021). NAC transcription factors *SNAC4* and *SNAC9* synergistically regulate tomato fruit ripening by affecting expression of genes involved in ethylene and abscisic acid metabolism and signal transduction. Postharvest Biol. Technol..

[63] Kou X.H., Zhao Y.N., Wu C., Jiang B.L., Zhang Z., Rathbun J.R., He Y.L., Xue Z.H. (2018). *SNAC4* and *SNAC9* transcription factors show contrasting effects on tomato carotenoids biosynthesis and softening. Postharvest Biol. Technol..

[64] Ma N.N., Feng H.L., Meng X., Li D., Yang D.Y., Wu C.G., Meng Q.W. (2014). Overexpression of tomato *SlNAC1* transcription factor alters fruit pigmentation and softening. BMC Plant Biol..

[65] Meng C., Yang D.Y., Ma X.C., Zhao W.Y., Liang X.Q., Ma N.N., Meng Q.W. (2016). Suppression of tomato *SlNAC1* transcription factor delays fruit ripening. J. Plant Physiol..

[66] Zhu M.K., Chen G.P., Zhou S., Tu Y., Wang Y., Dong T.T., Hu Z.L. (2014). A new tomato NAC (NAM/ATAF1/2/CUC2) transcription factor, SlNAC4, functions as a positive regulator of fruit ripening and carotenoid accumulation. Plant Cell Physiol..

[67] Jian W., Zheng Y.X., Yu T.T., Cao H.H., Chen Y., Cui Q.Y., Xu C., Li Z.G. (2021). SlNAC6, A NAC transcription factor, is involved in drought stress response and reproductive process in tomato. J. Plant Physiol..

[68] Zhu M.K., Hu Z.L., Zhou S., Wang L.L., Dong T.T., Pan Y., Chen G.P. (2014). Molecular characterization of six tissue-specific or stress-inducible genes of NAC transcription factor family in tomato (*Solanum lycopersicum*). J. Plant Growth Regul..

[69] Shahnejat-Bushehri S., Allu A.D., Mehterov N., Thirumalaikumar V.P., Alseekh S., Fernie A.R., Mueller-Roeber B., Balazadeh S. (2017). *Arabidopsis* NAC transcription factor JUNGBRUNNEN1 exerts conserved control over gibberellin and brassinosteroid metabolism and signaling genes in tomato. Front. Plant Sci..

[70] Gao Y., Fan Z.Q., Zhang Q., Li H.L., Liu G.S., Jing Y., Zhang Y.P., Zhu B.Z., Zhu H.L., Chen J.Y. (2021). A tomato NAC transcription factor, SlNAM1, positively regulates ethylene biosynthesis and the onset of tomato fruit ripening. Plant J..

[71] Wang A.D., Xu K.N. (2012). Characterization of two orthologs of REVERSION-TO-ETHYLENE SENSITIVITY1 in apple. J. Mol. Biol. Res..

[72] Zhang Q.J., Li T., Zhang L.J., Dong W.X., Wang A.D. (2018). Expression analysis of NAC genes during the growth and ripening of apples. Hortic. Sci..

[73] Migicovsky Z., Yeats T.H., Watts S., Song J., Forney C.F., Burgher-MacLellan K., Somers D.J., Gong Y.H., Zhang Z.Q., Vrebalov J. (2021). Apple ripening is controlled by a NAC transcription factor. Front. Genet..

[74] Fu C.C., Chen H.J., Gao H.Y., Wang S.L., Wang N., Jin J.C., Lu Y., Yu Z.L., Ma Q., Han Y.C. (2021). Papaya CpMADS4 and CpNAC3 co-operatively regulate ethylene signal genes *CpERF9* and *CpEIL5* during fruit ripening. Postharvest Biol. Technol..

[75] Shan W., Kuang J.F., Chen L., Xie H., Peng H.H., Xiao Y.Y., Li X.P., Chen W.X., He Q.G., Chen J.Y. (2012). Molecular characterization of banana NAC transcription factors and their interactions with ethylene signalling component EIL during fruit ripening. J. Exp. Bot..

[76] Shan W., Kuang J.F., Wei W., Fan Z.Q., Deng W., Li Z.G., Bouzayen M., Pirrello J., Lu W.J., Chen J.Y. (2020). MaXB3 Modulates MaNAC2, MaACS1, and MaACO1 stability to repress ethylene biosynthesis during banana fruit ripening. Plant Physiol..

[77] Wei W., Yang Y.Y., Su X.G., Kuang J.F., Chen J.Y., Lu W.J., Shan W. (2021). MaRTH1 suppression of ethylene response during banana fruit ripening and is controlled by MaXB3-MaNAC2 regulatory module. Postharvest Biol. Technol..

[78] Li B., Fan R.Y., Yang Q.S., Hu C.H., Sheng O., Deng G.M., Dong T., Li C.Y., Peng X.X., Bi F.C. (2020). Genome-wide identification and characterization of the NAC transcription factor family in *Musa acuminata* and expression analysis during fruit ripening. Int. J. Mol. Sci..

[79] Yan H.L., Jiang G.X., Wu F.W., Li Z.W., Xiao L., Jiang Y.M., Duan X.W. (2021). Sulfoxidation regulation of transcription factor NAC42 influences its functions in relation to stress-induced fruit ripening in banana. J. Exp. Bot..

[80] Li F., Li J.J., Qian M., Han M.Y., Cao L.J., Liu H.K., Zhang D., Zhao C.P. (2016). Identification of peach NAP transcription factor genes and characterization of their expression in vegetative and reproductive organs during development and senescence. Front. Plant Sci..

[81] Tan Q.P., Li S., Zhang Y.Z., Chen M., Wen B.B., Jiang S., Chen X.D., Fu X.L., Li D.M., Wu H.Y. (2021). Chromosome-level genome assemblies of five Prunus species and genome-wide association studies for key agronomic traits in peach. Hortic. Res..

[82] Guo Z.H., Zhang Y.J., Yao J.L., Xie Z.H., Zhang Y.Y., Zhang S.L., Gu C. (2021). The NAM/ATAF1/2/CUC2 transcription factor PpNAC.A59 enhances *PpERF.A16* expression to promote ethylene biosynthesis during peach fruit ripening. Hortic. Res..

[83] Wu Y.Y., Liu X.F., Fu B.L., Zhang Q.Y., Tong Y., Wang J., Wang W.Q., Grierson D., Yin X.R. (2020). Methyl jasmonate enhances ethylene synthesis in kiwifruit by inducing *NAC* genes that activate *ACS1*. J. Agric. Food Chem..

[84] Nieuwenhuizen N.J., Chen X.Y., Pellan M., Zhang L., Guo L., Laing W.A., Schaffer R.J., Atkinson R.G., Allan A.C. (2021). Regulation of wound ethylene biosynthesis by NAC transcription factors in kiwifruit. BMC Plant Biol..

[85] Wang W.Q., Wang J., Wu Y.Y., Li D.W., Allan A.C., Yin X.R. (2020). Genome-wide analysis of coding and non-coding RNA reveals a conserved miR164-*NAC* regulatory pathway for fruit ripening. N. Phytol..

[86] Fu B.L., Wang W.Q., Liu X.F., Duan X.W., Allan A.C., Grierson D., Yin X.R. (2021). An ethylene-hypersensitive methionine sulfoxide reductase regulated by NAC transcription factors increases methionine pool size and ethylene production during kiwifruit ripening. New Phytol..

[87] Moyano E., Martinez-Rivas F.J., Blanco-Portales R., Molina-Hidalgo F.J., Ric-Varas P., Matas-Arroyo A.J., Caballero J.L., Munoz-Blanco J., Rodriguez-Franco A. (2018). Genome-wide analysis of the NAC transcription factor family and their expression during the development and ripening of the *Fragaria × ananassa* fruits. PLoS ONE.

[88] Martín-Pizarro C., Vallarino J.G., Osorio S., Meco V., Urrutia M., Pillet J., Casanal A., Merchante C., Amaya I., Willmitzer L. (2021). The NAC transcription factor FaRIF controls fruit ripening in strawberry. Plant Cell.

[89] Carrasco-Orellana C., Stappung Y., Mendez-Yanez A., Allan A.C., Espley R.V., Plunkett B.J., Moya-Leon M.A., Herrera R. (2018). Characterization of a ripening-related transcription factor FcNAC1 from Fragaria chiloensis fruit. Sci. Rep..

[90] Zhu F., Luo T., Liu C.Y., Wang Y., Zheng L., Xiao X., Zhang M.F., Yang H.B., Yang W., Xu R.W. (2020). A NAC transcription factor and its interaction protein hinder abscisic acid biosynthesis by synergistically repressing *NCED5* in *Citrus reticulata*. J. Exp. Bot..

[91] Rios P., Argyris J., Vegas J., Leida C., Kenigswald M., Tzuri G., Troadec C., Bendahmane A., Katzir N., Pico B. (2017). *ETHQV6.3* is involved in melon climacteric fruit ripening and is encoded by a NAC domain transcription factor. Plant J..

[92] Tranbarger T.J., Fooyontphanich K., Roongsattham P., Pizot M., Collin M., Jantasuriyarat C., Suraninpong P., Tragoonrung S., Dussert S., Verdeil J.L. (2017). Transcriptome analysis of cell wall and NAC domain transcription factor genes during *Elaeis guineensis* fruit ripening: Evidence for widespread conservation within monocot and eudicot lineages. Front. Plant Sci..

[93] Huang G.H., Li T., Li X.Y., Tan D.M., Jiang Z.Y., Wei Y., Li J.C., Wang A.D. (2014). Comparative transcriptome analysis of climacteric fruit of Chinese pear (*Pyrus ussuriensis*) reveals new insights into fruit ripening. PLoS ONE.

[94] Li X., Xu C.J., Korban S.S., Chen K.S. (2010). Regulatory mechanisms of textural changes in ripening fruits. Crit. Rev. Plant Sci..

[95] Brummell D.A. (2006). Cell wall disassembly in ripening fruit. Funct. Plant Biol..

[96] Goulao L.F., Oliveira C.M. (2007). Cell wall modifications during fruit ripening: When a fruit is not the fruit. Trends Food Sci. Technol..

[97] Tucker G., Yin X.R., Zhang A.D., Wang M.M., Zhu Q.G., Liu X.F., Xie X.L., Chen K.S., Grierson D. (2017). Ethylene and fruit softening. Food Qual. Saf..

[98] Pose S., Paniagua C., Matas A.J., Gunning A.P., Morris V.J., Quesada M.A., Mercado J.A. (2019). A nanostructural view of the cell wall disassembly process during fruit ripening and postharvest storage by atomic force microscopy. Trends Food Sci. Technol..

[99] Brummell D.A., Harpster M.H., Civello P.M., Palys J.M., Bennett A.B., Dunsmuir P. (1999). Modification of expansin protein abundance in tomato fruit alters softening and cell wall polymer metabolism during ripening. Plant Cell.

[100] Rose J.K.C., Bennett A.B. (1999). Cooperative disassembly of the cellulose-xyloglucan network of plant cell walls: Parallels between cell expansion and fruit ripening. Trends Plant Sci..

[101] Brummell D.A., Harpster M.H. (2001). Cell wall metabolism in fruit softening and quality and its manipulation in transgenic plants. Plant Mol. Biol..

[102] Wang D.D., Yeats T.H., Uluisik S., Rose J.K.C., Seymour G.B. (2018). Fruit softening: Revisiting the role of pectin. Trends Plant Sci..

[103] Jing L., Li J., Song Y.Z., Zhang J.Y., Chen Q., Han Q.Q. (2018). Characterization of a potential ripening regulator, *SlNAC3*, from *Solanum Lycopersicum*. Open Life Sci..

[104] Gao Y., Zhang Y.P., Fan Z.Q., Jing Y., Chen J.Y., Grierson D., Yang R., Fu D.Q. (2021). Mutagenesis of *SlNAC4* by CRISPR/Cas9 alters gene expression and softening of ripening tomato fruit. Veg. Res..

[105] Xu Q., Wang W.Q., Zeng J.K., Zhang J., Grierson D., Li X., Yin X.R., Chen K.S. (2015). A NAC transcription factor, *EjNAC1*, affects lignification of loquat fruit by regulating lignin. Postharvest Biol. Technol..

[106] Ge H., Zhang J., Zhang Y.J., Li X., Yin X.R., Grierson D., Chen K.S. (2017). EjNAC3 transcriptionally regulates chilling-induced lignification of loquat fruit via physical interaction with an atypical CAD-like gene. J. Exp. Bot..

[107] Zhang Q., Wang L.H., Wang Z.T., Zhang R.T., Liu P., Liu M.J., Liu Z.G., Zhao Z.H., Wang L.L., Chen X. (2021). The regulation of cell wall lignification and lignin biosynthesis during pigmentation of winter jujube. Hortic. Res..

[108] Li M., Hou L., Liu S.S., Zhang C.X., Yang W.C., Pang X.M., Li Y.Y. (2021). Genome-wide identification and expression analysis of NAC transcription factors in *Ziziphus jujuba* Mill. reveal their putative regulatory effects on tissue senescence and abiotic stress responses. Ind. Crops Prod..

[109] Kuai B., Chen J.Y., Hortensteiner S. (2018). The biochemistry and molecular biology of chlorophyll breakdown. J. Exp. Bot..

[110] Luan Y.T., Fu X.M., Lu P.J., Grierson D., Xu C.J. (2020). Molecular mechanisms determining the differential accumulation of carotenoids in plant species and varieties. Crit. Rev. Plant Sci..

[111] Sun T.H., Yuan H., Cao H.B., Yazdani M., Tadmor Y., Li L. (2018). Carotenoid metabolism in plants: The role of plastids. Mol. Plant..

[112] Jaakola L. (2013). New insights into the regulation of anthocyanin biosynthesis in fruits. Trends Plant Sci..

[113] Sun Q.G., Jiang S.H., Zhang T.L., Xu H.F., Fang H.C., Zhang J., Su M.Y., Wang Y.C., Zhang Z.Y., Wang N. (2019). Apple NAC transcription factor MdNAC52 regulates biosynthesis of anthocyanin and proanthocyanidin through MdMYB9 and MdMYB11. Plant Sci..

[114] Zhang S.Y., Chen Y.X., Zhao L.L., Li C.Q., Yu J.Y., Li T.T., Yang W.Y., Zhang S.N., Su H.Y., Wang L. (2020). A novel NAC transcription factor, MdNAC42, regulates anthocyanin accumulation in red-fleshed apple by interacting with MdMYB10. Tree Physiol..

[115] Sun Q.G., Jiang S.H., Fang H.C., Zhang T.L., Wang N., Chen X.S. (2019). Cloning of *MdNAC9* and functional of its regulation on flavonol synthesis. Acta Hortic. Sin..

[116] Fu C.C., Han Y.C., Fan Z.Q., Chen J.Y., Chen W.X., Lu W.J., Kuang J.F. (2016). The papaya transcription factor CpNAC1 modulates carotenoid biosynthesis through activating phytoene desaturase genes *CpPDS2/4* during fruit ripening. J. Agric. Food Chem..

[117] Fu C.C., Han Y.C., Kuang J.F., Chen J.Y., Lu W.J. (2017). Papaya CpEIN3a and CpNAC2 co-operatively regulate carotenoid biosynthesis-related genes *CpPDS2/4*, *CpLCY-e* and *CpCHY-b* during fruit ripening. Plant Cell Physiol..

[118] Zhou H., Kui L.W., Wang H.L., Gu C., Dare A.P., Espley R.V., He H.P., Allan A.C., Han Y.P. (2015). Molecular genetics of blood-fleshed peach reveals activation of anthocyanin biosynthesis by NAC transcription factors. Plant J..

[119] Pirona R., Eduardo I., Pacheco I., Linge C.D., Miculan M., Verde I., Tartarini S., Dondini L., Pea G., Bassi D. (2013). Fine mapping and identification of a candidate gene for a major locus controlling maturity date in peach. BMC Plant Biol..

[120] Nunez-Lillo G., Cifuentes-Esquivel A., Troggio M., Micheletti D., Infante R., Campos-Vargas R., Orellana A., Blanco-Herrera F., Meneses C. (2015). Identification of candidate genes associated with mealiness and maturity date in peach [*Prunus persica* (L.) Batsch] using QTL analysis and deep sequencing. Tree Genet. Genomes.

[121] Eduardo I., Picanol R., Rojas E., Batlle I., Howad W., Aranzana M.J., Arus P. (2015). Mapping of a major gene for the slow ripening character in peach: Co-location with the maturity date gene and development of a candidate gene-based diagnostic marker for its selection. Euphytica.

[122] Balogh E., Halasz J., Szani Z., Hegedus A. (2018). Correspondence between maturity date and molecular variations in a *NAC* transcription factor of diploid and polyploid *Prunus* species. Turk. J. Agric. For..

[123] Qin J., Yu F., Liu L., Zhu T.T., Chen W., Cao S.F., Yang Z.F., Shi L.Y. (2021). Cloning of peach *PpNAC19* and its regulation on *PpCCD4* promoter activity. J. Nucl. Agric. Sci..

[124] Lin Y.X., Jiang L.Y., Chen Q., Li Y.L., Zhang Y.T., Luo Y., Zhang Y., Sun B., Wang X.R., Tang H.R. (2018). Comparative transcriptome profiling analysis of red- and white-fleshed strawberry (*Fragaria × ananassa*) provides new insight into the regulation of the anthocyanin pathway. Plant Cell Physiol..

[125] Gong J.L., Zeng Y.L., Meng Q.N., Guan Y.J., Li C.Y., Yang H.B., Zhang Y.Z., Ampomah-Dwamena C., Liu P., Chen C.W. (2021). Red light-induced kumquat fruit coloration is attributable to increased carotenoid metabolism regulated by FcrNAC22. J. Exp. Bot..

[126] Jiang G.X., Li Z.W., Song Y.B., Zhu H., Lin S., Huang R.M., Jiang Y.M., Duan X.W. (2019). LcNAC13 physically interacts with LcR1MYB1 to coregulate anthocyanin biosynthesis-related genes during litchi fruit ripening. Biomolecules.

[127] Ahmad M., Yan X.H., Li J.Z., Yang Q.S., Jamil W., Teng Y.W., Bai S.L. (2018). Genome wide identification and predicted functional analyses of NAC transcription factors in Asian pears. BMC Plant Biol..

[128] Batista-Silva W., Nascimento V.L., Medeiros D.B., Nunes-Nesi A., Ribeiro D.M., Zsogon A., Araujo W.L. (2018). Modifications in organic acid profiles during fruit development and ripening: Correlation or causation?. Front. Plant Sci..

[129] Quinet M., Angosto T., Yuste-Lisbona F.J., Blanchard-Gros R., Bigot S., Martinez J.P., Lutts S. (2019). Tomato fruit development and metabolism. Front. Plant Sci..

[130] Song J., Forney C.F. (2008). Flavour volatile production and regulation in fruit. Can. J. Plant Sci..

[131] Defilippi B.G., Manriquez D., Luengwilai K., Gonzalez-Aguero M. (2009). Aroma volatiles: Biosynthesis and mechanisms of modulation during fruit ripening. Adv. Bot. Res..

[132] Wang L.B., Baldwin E.A., Bai J.H. (2016). Recent advance in aromatic volatile research in tomato fruit: The metabolisms and regulations. Food Bioprocess Technol..

[133] Ma X.M., Zhang Y.J., Tureckova V., Xue G.P., Fernie A.R., Mueller-Roeber B., Balazadeh S. (2018). The NAC transcription factor SlNAP2 regulates leaf senescence and fruit yield in tomato. Plant Physiol..

[134] Ma X.M., Balazadeh S., Mueller-Roeber B. (2019). Tomato fruit ripening factor NOR controls leaf senescence. J. Exp. Bot..

[135] Lira B.S., Gramegna G., Trench B.A., Alves F.R.R., Silva E.M., Silva G.F.F., Thirumalaikumar V.P., Lupi A.C.D., Demarco D., Purgatto E. (2017). Manipulation of a senescence-associated gene improves fleshy fruit yield. Plant Physiol..

[136] Song Z.Y., Qin J.J., Zheng Q.L., Ding X.C., Chen W.X., Lu W.J., Li X.P., Zhu X.Y. (2019). The Involvement of the banana F-Box protein MaEBF1 in regulating chilling-inhibited starch degradation through interaction with a MaNAC67-like protein. Biomolecules.

[137] Lv X., Lan S.R., Guy K.M., Yang J.H., Zhang M.F., Hu Z.Y. (2016). Global expressions landscape of NAC transcription factor family and their responses to abiotic stresses in *Citrullus lanatus*. Sci. Rep..

[138] Wang J.F., Wang Y.P., Zhang J., Ren Y., Li M.Y., Tian S.W., Yu Y.T., Zuo Y., Gong G.Y., Zhang H.Y. (2021). The NAC transcription factor *ClNAC68* positively regulates sugar content and seed development in watermelon by repressing *ClINV* and *ClGH3.6*. Hortic. Res..

[139] Li S.J., Yin X.R., Wang W.L., Liu X.F., Zhang B., Chen K.S. (2017). Citrus CitNAC62 cooperates with CitWRKY1 to participate in citric acid degradation via up-regulation of *CitAco3*. J. Exp. Bot..

[140] Min T., Wang M.M., Wang H.X., Liu X.F., Fang F., Grierson D., Yin X.R., Chen K.S. (2015). Isolation and expression of *NAC* genes during persimmon fruit postharvest astringency removal. Int. J. Mol. Sci..

[141] Jin R., Zhu Q.G., Shen X.Y., Wang M.M., Jamil W., Grierson D., Yin X.R., Chen K.S. (2018). *DkNAC7*, a novel high-CO_2_/hypoxia-induced NAC transcription factor, regulates persimmon fruit de-astringency. PLoS ONE.

[142] Jamil W., Wu W., Ahmad M., Zhu Q.G., Liu X.F., Jin R., Yin X.R. (2019). High-CO_2_/hypoxia-modulated NAC transcription factors involved in de-astringency of persimmon fruit. Sci. Hortic..

[143] Nieuwenhuizen N.J., Chen X.Y., Wang M.Y., Matich A.J., Perez R.L., Allan A.C., Green S.A., Atkinson R.G. (2015). Natural variation in monoterpene synthesis in kiwifruit: Transcriptional regulation of terpene synthases by NAC and ETHYLENE-INSENSITIVE3-like transcription factors. Plant Physiol..

[144] Cao X.M., Wei C.Y., Duan W.Y., Gao Y., Kuang J.F., Liu M.C., Chen K.S., Klee H., Zhang B. (2021). Transcriptional and epigenetic analysis reveals that NAC transcription factors regulate fruit flavor ester biosynthesis. Plant J..

[145] Pandita V.K., Nagarajan S. (2001). Fruit maturity and post harvest ripening affecting chilli seed quality and field emergence. Seed Res..

[146] Yogeesha H.S., Vasugi C., Somashekhar K.B., Naik L.B. (2013). Papaya (*Carica papaya*) seed quality as influenced by stage of fruit harvest, postharvest ripening and seed extraction. Indian J. Agric. Sci..

[147] Vinod K., Tomar B.S., Kaddi G., Kumar S. (2014). Effect of stage of harvest and post-harvest ripening of fruits on hybrid seed yield and quality in pumpkin (*Cucurbita moschata*). Indian J. Agric. Sci..

[148] Cremon T., Dresch D.M., Scalon S.P.Q., Masetto T.E. (2018). Drying and reduction in sensitivity to desiccation of seeds of *Alibertia edulis*: The influence of fruit ripening stage. An. Acad. Bras. Cienc..

[149] Han Q.Q., Song Y.Z., Zhang J.Y., Liu L.F. (2014). Studies on the role of the *SlNAC3* gene in regulating seed development in tomato (*Solanum lycopersicum*). J. Hortic. Sci. Biotechnol..

[150] Kunieda T., Mitsuda N., Ohme-Takagi M., Takeda S., Aida M., Tasaka M., Kondo M., Nishimura M., Hara-Nishimura I. (2008). NAC family proteins NARS1/NAC2 and NARS2/NAM in the outer integument regulate embryogenesis in *Arabidopsis*. Plant Cell.

[151] Mathew I.E., Das S., Mahto A., Agarwal P. (2016). Three rice NAC transcription factors heteromerize and are associated with seed size. Front. Plant Sci..

[152] Ren Y., Huang Z.Q., Jiang H., Wang Z., Wu F.S., Xiong Y.F., Yao J.L. (2021). A heat stress responsive NAC transcription factor heterodimer plays key roles in rice grain filling. J. Exp. Bot..

[153] Zhang S.L., Dong R.Z., Wang Y.W., Li X.M., Ji M.M., Wang X.P. (2021). NAC domain gene VvNAC26 interacts with VvMADS9 and influences seed and fruit development. Plant Physiol. Biochem..

[154] Meng Q.C., Zhang C.H., Gai J.Y., Yu D.Y. (2007). Molecular cloning, sequence characterization and tissue-specific expression of six NAC-like genes in soybean (*Glycine max* (L.) Merr.). J. Plant Physiol..

[155] Garcia-Gomez B.E., Salazar J.A., Dondini L., Martinez-Gomez P., Ruiz D. (2019). Identification of QTLs linked to fruit quality traits in apricot (*Prunus armeniaca* L.) and biological validation through gene expression analysis using qPCR. Mol. Breed..

[156] Xu W.Y., Chen C., Gou N.N., Huang M.Z., Wuyun T.N., Zhu G.P., Zhao H., Liu H.M., Wang L. (2021). Genome-wide identification and expression analysis of NAC transcription factor family genes during fruit and kernel development in Siberian Apricot. J. Am. Soc. Hortic. Sci..

[157] Tian S.P., Qin G.Z., Li B.Q. (2013). Reactive oxygen species involved in regulating fruit senescence and fungal pathogenicity. Plant Mol. Biol..

[158] Xu X.B., Yin L.L., Ying Q.C., Song H.M., Xue D.W., Lai T.F., Xu M.J., Shen B., Wang H.Z., Shi X.Q. (2013). High-throughput sequencing and degradome analysis identify miRNAs and their targets involved in fruit senescence of *Fragaria ananassa*. PLoS ONE.

[159] Li J., Lai T., Song H., Xu X. (2017). MiR164 is involved in delaying senescence of strawberry (*Fragaria ananassa*) fruit by negatively regulating *NAC* transcription factor genes under low temperature. Russ. J. Plant Physiol..

[160] Wang Y.X., Li W.S., Chang H., Zhou J.H., Luo Y.B., Zhang K.C., Zuo J.H., Wang B.G. (2020). SRNAome and transcriptome analysis provide insight into strawberry fruit ripening. Genomics.

[161] Liu Y.Z., Baig M.N.R., Fan R., Ye J.L., Cao Y.C., Deng X.X. (2009). Identification and expression pattern of a novel NAM, ATAF, and CUC-like gene from *Citrus sinensis* Osbeck. Plant Mol. Biol. Rep..

[162] Jiang G.X., Yan H.L., Wu F.W., Zhang D.D., Zeng W., Qu H.X., Chen F., Tan L., Duan X.W., Jiang Y.M. (2017). Litchi fruit LcNAC1 is a target of LcMYC2 and regulator of fruit senescence through its interaction with LcWRKY1. Plant Cell Physiol..

[163] Jiang G.X., Xiao L., Yan H.L., Zhang D.D., Wu F.W., Liu X.C., Su X.G., Dong X.H., Wang J.S., Duan X.W. (2017). Redox regulation of methionine in calmodulin affects the activity levels of senescence-related transcription factors in litchi. Biochim. Biophys. Acta-Gen. Subj..

[164] Kou X.H., Watkins C.B., Gan S.S. (2012). *Arabidopsis AtNAP* regulates fruit senescence. J. Exp. Bot..

[165] Mizzotti C., Rotasperti L., Moretto M., Tadini L., Resentini F., Galliani B.M., Galbiati M., Engelen K., Pesaresi P., Masiero S. (2018). Time-course transcriptome analysis of Arabidopsis siliques discloses genes essential for fruit development and maturation. Plant Physiol..

[166] Guo Y.F., Gan S.S. (2006). AtNAP, a NAC family transcription factor, has an important role in leaf senescence. Plant J..

